# The general control nonderepressible-2 kinase mediates stress response and longevity induced by target of rapamycin inactivation in *Caenorhabditis elegans*

**DOI:** 10.1111/acel.12101

**Published:** 2013-06-28

**Authors:** Aris Rousakis, Arsenios Vlassis, Anna Vlanti, Stefania Patera, George Thireos, Popi Syntichaki

**Affiliations:** 1 Biomedical Research Foundation of the Academy of Athens, Center of Basic Research IIAthens, 11527, Greece; 2 School of Medicine, University of AthensAthens, 11527, Greece; 3 Faculty of Biology, University of AthensAthens, 15701, Greece

**Keywords:** general control nonderepressible 2, aging, target of rapamycin, *Caenorhabditis elegans*, PHA-4

## Abstract

The general control nonderepressible 2 (GCN2) kinase is a nutrient-sensing pathway that responds to amino acids deficiency and induces a genetic program to effectively maintain cellular homeostasis. Here we established the conserved role of *Caenorhabditis elegans* GCN-2 under amino acid limitation as a translation initiation factor 2 (eIF2) kinase. Using a combination of genetic and molecular approaches, we showed that GCN-2 kinase activity plays a central role in survival under nutrient stress and mediates lifespan extension conferred by dietary restriction (DR) or inhibition of the major nutrient-sensing pathway, the target of rapamycin (TOR). We also demonstrated that the GCN-2 and TOR signaling pathways converge on the PHA-4/FoxA transcription factor and its downstream target genes to ensure survival of the whole organism under a multitude of stress conditions, such as nutrient scarcity or environmental stresses. This is one step forward in the understanding of evolutionary conserved mechanisms that confer longevity and healthspan.

## Introduction

The ability of most organisms to survive relies on their capability to rapidly trigger a coordinated systemic response upon nutrient or environmental stresses. In eukaryotes, such a stress response involves the inhibition of global protein synthesis, with a concomitant reprogramming of gene expression, which allow cells to conserve resources, maintain cellular homeostasis, and effectively deal with stress (Spriggs *et al*., 2010). Inhibition of protein synthesis is attained through phosphorylation of the alpha subunit of the translation initiation factor 2 (eIF2α) by specific protein kinases, each activated by different stress signals (Wek *et al*., 2006). General control nonderepressible 2 is the only eIF2α kinase conserved from yeast to mammals that regulates amino acid transport and metabolism in response to nutrient depletion (Hinnebusch, 2005). GCN2 can also be activated by other stresses, such as UV irradiation, and has been shown to regulate many other vital cellular processes in mammals, such as lipid metabolism, oxidative stress resistance, feeding behavior, NF-kB signaling upon UV radiation, synaptic plasticity, and memory (Harding *et al*., 2003; Costa-Mattioli *et al*., 2009).

Phosphorylation of eIF2α under stress results in inhibition of global protein synthesis, which is accompanied by favored translation of specific mRNAs that adapt the organism to stress. These mRNAs include potent transcription factors such as GCN4 in yeast (Natarajan *et al*., 2001) and ATF4 in mammals (Harding *et al*., 2003). The proposed model for the translation of these mRNAs, under amino acid limitation, involves the upstream open reading frames (uORFs) that are located in their 5′-UTR (Tzamarias & Thireos, 1988; Vattem & Wek, 2004). These uORFs are preferentially translated in the nonstressed condition, leading to synthesis of incorrect peptides and precluding translation of the authentic gene initiation site (Hinnebusch, 2005). Under stress, the inhibitory effect of eIF2α phosphorylation to the levels of the active ternary complex increases the probability that ribosomal scanning will bypass the uORFs, and translation re-initiation will occur at the canonical *gcn4* or *atf4* initiation site.

The mechanisms of translational and transcriptional reprogramming under stress have emerged as important mediators of lifespan extension and stress resistance in *Caenorhabditis elegans* (Curran & Ruvkun, 2007; Hansen *et al*., 2007; Syntichaki *et al*., 2007; Rogers *et al*., 2011). Consistent with this, aging in many organisms is modulated by conserved signaling pathways that affect numerous cellular processes, involving regulation of translation. One such pathway is that of the target of rapamycin (TOR) kinase, which responds to hormonal, nutrient, and environmental stress signals to regulate growth, differentiation, and metabolism of eukaryotes (Cornu *et al*., 2013). The TOR pathway has been identified as a mediator of DR-induced longevity in yeast, worms, flies, and mice (Kapahi *et al*., 2010), whereas differential expression of TOR signaling has been recently associated with advancing age in human populations (Harries *et al*., 2012; Passtoors *et al*., 2013). The downstream targets of TOR signaling involve mRNA translation, ribosome synthesis, transcription, stress response, metabolism, and autophagy. All these cellular processes have been linked to the longevity effects of TOR disruption in multiple species.

Despite the well-established link between nutrient limitation and lifespan in many eukaryotes, the interplay between the two major nutrient-sensing pathways, GCN2 and TOR, in longevity still remains unclear. Reduced availability of nutrients such as nitrogen, carbon, or amino acids inhibits TOR and induces autophagy in yeast and mammals (Jung *et al*., 2010). Also in mice, the impaired amino acid uptake in intestinal cells leads to increased phosphorylation of eIF2α, suggesting activation of the GCN2 pathway and reduced TOR signaling (Broer *et al*., 2011). Rapamycin (a TOR-specific inhibitor) has been shown to derepress translation of yeast GCN4 through activation of GCN2 (Cherkasova & Hinnebusch, 2003; Kubota *et al*., 2003). Genetic evidence in yeast suggests that DR, TOR inhibition or depletion of the 60S ribosomal subunits mediate replicative lifespan partially through the activation of the GCN4 transcription factor (Steffen *et al*., 2008). Recently, a role of GCN2 in the aging process was suggested in mice, where the absence of GCN2 affects macronutrient selection changes during aging (Maurin *et al*., 2012). Herein, we have established the conserved function of the GCN-2 kinase in *C. elegans* under amino acid limitation, and we showed that loss of GCN-2 activity is not required for normal lifespan, but affects the lifespan of nutrient-sensitized worms. We revealed that GCN-2 signaling positively regulates the induction of PHA-4/FoxA transcription factor under nutrient or oxidative stress, as part of the adaptive response that ensures stress survival and longevity.

## Results

### A GCN-2-dependent phosphorylation of eIF2α under amino acid limitation

**Figure 1:**
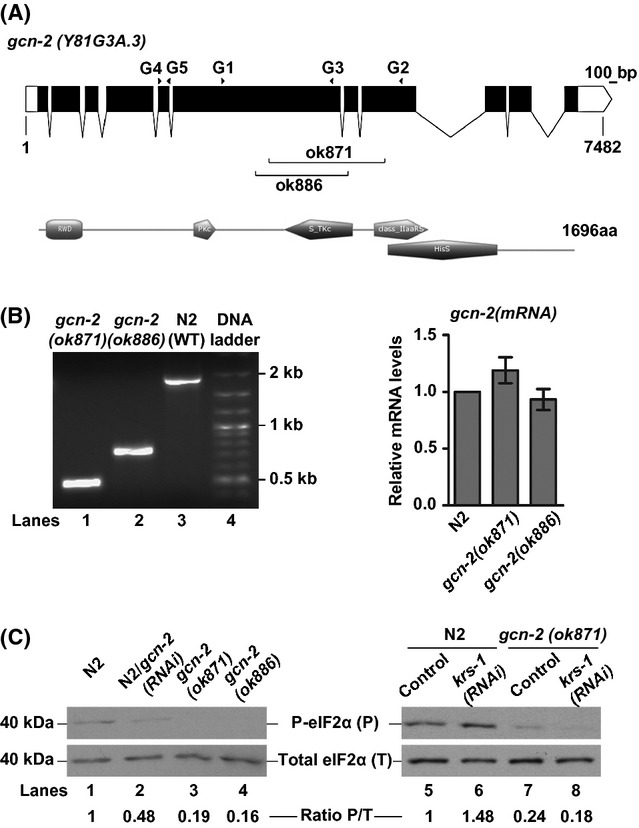
Conservation in gene structure and function of *Caenorhabditis elegans* *gcn-2*. (A) Gene structure and predicted protein domains of GCN-2, designed using the Prosite MyDomains (http://prosite.expasy.org/mydomains/): Black boxes represent exons linked by lines corresponding to introns, and white boxes indicate the 5′ and 3′ UTR found in a second alternative isoform (http://www.wormbase.org). The graphic was created using the Exon-Intron Graphic Maker (http://wormweb.org/exonintron). Branches point to the sequences deleted in the two alleles *ok871* and *ok886*. Black arrowheads indicate the position of primers used in this study (Table S2). (B) Reverse transcription (RT)–PCR analysis with primers G1/G2 (shown in A pane) and qRT-PCR analysis with primers G4/G5 (shown in A pane). (C) Western blot analysis showing the levels of phosphorylated (P-eIF2α) normalized by the total amount of eIF2α, in whole worm extracts. Basal levels of P-eIF2α under well-fed conditions in untreated or *gcn-2(RNAi)*-treated N2 and *gcn-2* mutants (left pane). Induced levels of P-eIF2α under amino acid limitation in *krs-1(RNAi)* treated worms (right pane).

In *C. elegans*, the sole homolog of yeast/mammalian GCN2 is encoded by the gene Y81G3A.3 (*gcn-2*) and phosphorylates the eIF2α subunit at the putative phosphorylation site Ser49 (Nukazuka *et al*., 2008). Lately, it was shown that GCN-2 is required for the induced phospho-eIF2α levels during mitochondrial or osmotic stress (Baker *et al*., 2012; Lee & Strange, 2012), but its function during nutrient deprivation or other stresses has not been established. The predicted GCN-2 protein of 1696 amino acids shares 24.2% identity (40.7% similarity) with human HsGCN2 and 21% identity (35.3% similarity) with yeast ScGCN2 (EMBOSS Align-EMBL/EBI), having all the functional domains that characterize the kinase across species (Fig. 1A). To investigate the function of GCN-2, we used the existing *gcn-2* mutants (*ok871* and *ok886*), both of which have an in-frame deletion (Wormbase WS230), lacking part of the internal coding sequence (Fig. 1A). In both *gcn-2* alleles, we detected a truncated mRNA transcript (lanes 1 and 2 in Fig. 1B, left pane) expressed at the same levels as the wild-type (N2) transcript (Fig. 1B, right pane). By measuring the basal levels of eIF2α phosphorylation in whole protein extracts of N2 worms subjected to *gcn-2(RNAi)* (lane 2 in Fig. 1C) or of each *gcn-2* mutant (lanes 3–4 in Fig. 1C), compared to untreated animals (lane 1 in Fig. 1C), we verified that both *ok871* and *ok886* are loss-of-function alleles of *gcn-2*.

To determine whether worm GCN-2 kinase responds to amino acid limitation, we raised worms on plates seeded with bacteria expressing dsRNA against *krs-1* gene, the worm lysil-tRNA synthetase. Aminoacyl-tRNA synthetases (AARSs) catalyze the ligation of specific amino acids to their cognate tRNAs and are important for cellular protein synthesis. Changes in the levels of AARSs affect the levels of uncharged tRNAs, and this consists the major signal for GCN2 activation and eIF2α phosphorylation in other organisms. We found that worms grown on bacteria expressing dsRNA for *krs-1* either arrested in early larval stages or became adults with low brood size (∼20% of the normal), depending on the starting time of RNAi treatment (eggs or L3–L4 stage, respectively). In such *krs-1(RNAi)*-fed adults, we monitored an increase (∼50%) of the phospho-eIF2α levels (lane 6 in Fig. 1C), compared to untreated control animals (lane 5 in Fig. 1C). We observed the same level of induction using RNAi for *lrs-1*, encoding a leucyl-tRNA synthetase (lanes 1–2 in Fig. S1). Moreover, this increase depends on GCN-2 activity (lanes 7–8 in Fig. 1C and lanes 3–4 in Fig. S1). All these are consistent with the conserved role of worm GCN-2 as an eIF2α kinase under amino acid limitation conditions.

### Favored translation of *atf-5* during amino acid limitation

**Figure 2:**
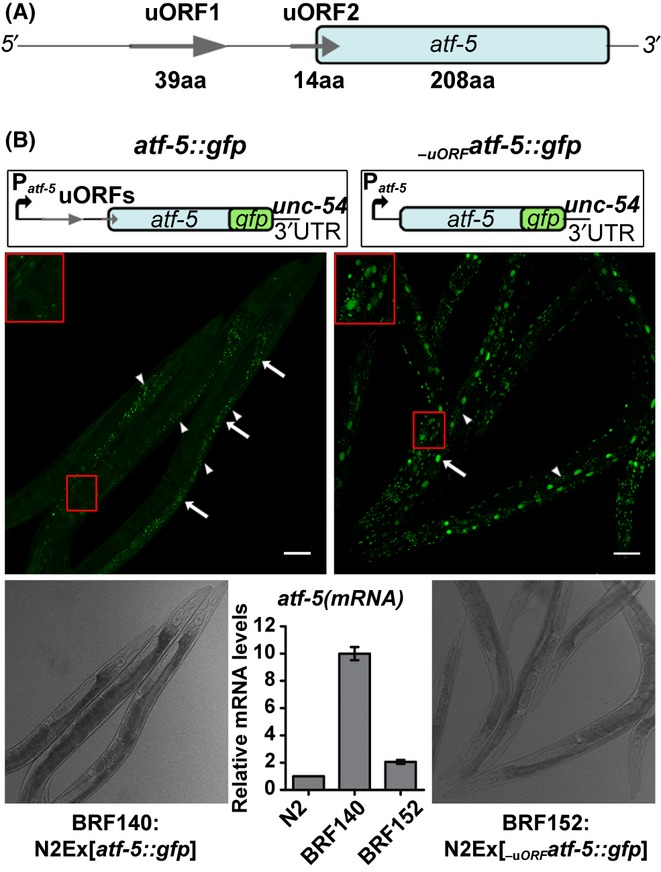
Translational control of *atf-5* gene expression. (A) Schema of uORFs within the *atf-5* 5′-UTR. The amino acid length of the predicted translated uORF and the coding sequence of *atf-5* gene are shown. (B) Confocal and bright field images of 1-day-old transgenic worms expressing the translational fusion of the intact *atf-5* (left box) or the uORF-less *atf-5* transgene (right box) under normal feeding conditions. A larger magnification of the area in the red box is shown on the top left. White arrows indicate fluorescent nuclei, and white arrowheads show regions of autofluorescence. All images were taken at 20× magnification under the same microscopy settings (scale bar: 50 μm). The levels of *atf-5* mRNA in N2, BRF140, and BRF152 worms, normalized to *ama-1(mRNA)*, were quantified using qRT–PCR.

Phosphorylation of eIF2α in yeast and mammals has two consequences: inhibition of global protein synthesis and induction of specific mRNA translation. In *C. elegans,* it has been shown that knockdown of several genes encoding AARSs reduces [^35^S]methionine incorporation and protein synthesis rate (Anderson *et al*., 2009), in agreement with the increased phospho-eIF2α levels shown in Fig. 1C (compare lanes 5 and 6). Therefore, we tested whether phosphorylation of eIF2α under amino acid deprivation could also induce translation of specific mRNAs. The worm homolog of yeast GCN4 and the related mammalian ATF4 transcription factor is encoded by the *atf-5* (T04C10.4) gene, bearing two upstream ORFs (Fig. 2A). To evaluate the role of uORFs in *atf-5* mRNA translation, we created transgenic animals carrying either the intact *atf-5* gene including the two uORFs (BRF140) or the *atf-5* coding sequence lacking both uORFs (BRF152). In both cases, the transgenes were fused at C′-terminus with *gfp,* and their expression was driven by the *atf-5* promoter. BRF140 worms showed only a weak fluorescent signal with *atf-5::gfp* expression in the nucleus of few cells, varying between individuals (Fig. 2B). In contrast, BRF152 worms displayed a bright GFP signal in the nucleus of all cells (Fig. 2B). As the expression levels of *atf-5* mRNA are increased ∼10-fold in BRF140 and ∼twofold in BRF152 young adults, compared to endogenous mRNA (Fig. 2B), it becomes evident that the presence of the uORFs in the 5′ leader of *atf-5*mRNA is inhibitory for its translation.

**Figure 3:**
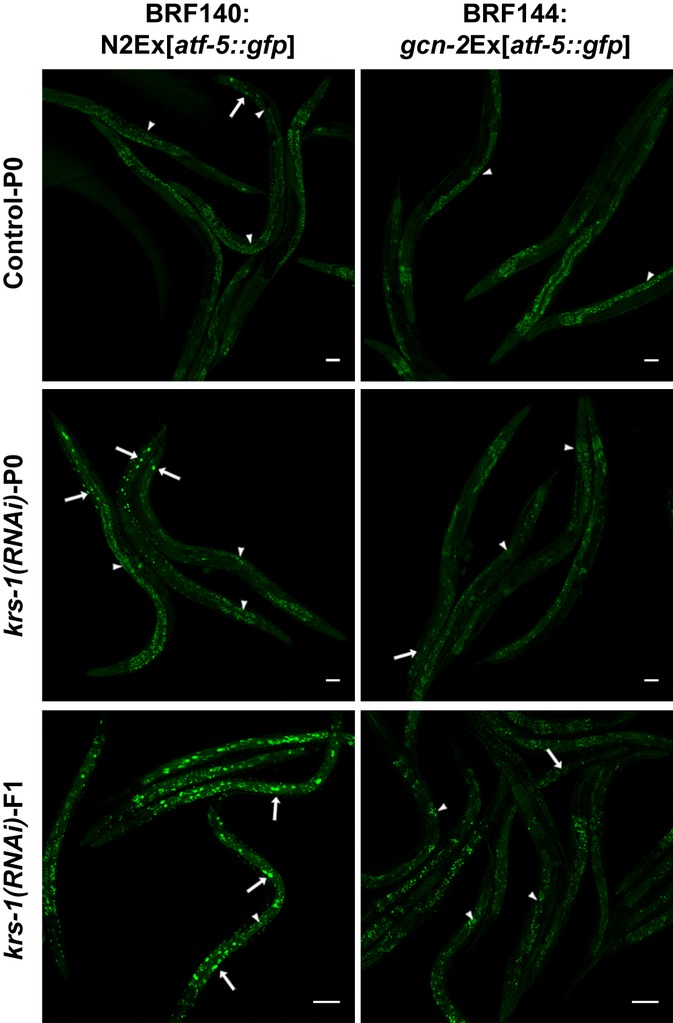
General control nonderepressible-2-dependent translational control of *atf-5* under amino acid limitation. Confocal images of N2 or *gcn-2(ok871)* worms, both carrying a translational fusion of *atf-5::gfp*, fed Control or *krs-1(RNAi)* expressing bacteria. The images show 1-day adults fed with each RNAi from eggs (P0) or their L3-arrested progeny (F1) in *krs-1(RNAi)* worms. White arrows indicate fluorescent nuclei; white arrowheads show regions of autofluorescence. All images were taken at 20× magnification under the same microscopy settings (scale bar: 50 μm).

The inhibitory effect of uORFs in the translation of the intact *atf-5* mRNA was also greatly ameliorated in response to amino acid limitation, as this was recapitulated through RNAi-mediated silencing of AARSs genes. Transgenic BRF140 worms subjected to *krs-1(RNAi)* showed enhanced fluorescent signal in neurons, hypodermis, muscles, and intestine (Fig. 3). The fluorescence intensity in the few L3-arrested progeny (F1) of the RNAi-treated animals (P0) almost attained the brightness of BRF152 worms. This enhancement was dependent on GCN-2 function as we showed by expressing the same intact *atf-5* transgene in the *gcn-2(ok871)* background (BRF144 in Fig. 3). Only the basal signal in varying cells and the autofluorescence of the intestine were observed. Similar results (Fig. S2) were obtained by inactivating an arginyl-tRNA synthetase (*rrt-1*) gene. The *gcn-2*-dependent induction of *atf-5::gfp* mirrored the *gcn-2*-dependent phosphorylation of eIF2α upon amino acid limitation. Consequently, direct inactivation of eIF2α by RNAi, instead of phosphorylation, bypasses the requirement for *gcn-2* resulting in upregulation of *atf-5::gfp* transgene in both N2 and *gcn-2* worms (Fig. S2). We also observed a *gcn-2*-independent induction of *atf-5::gfp* transgene after treatment of worms with a potent inducer of ER stress, tunicamycin (Fig. S2). Thus, the two uORFs direct the translational activation of the *C. elegans atf-5* gene under nutrient or other stresses, in agreement with the yeast *gcn4* and mammalian *atf4* homologs.

### General control nonderepressible-2 signaling influences the longevity of nutrient-sensitized worms

**Figure 4:**
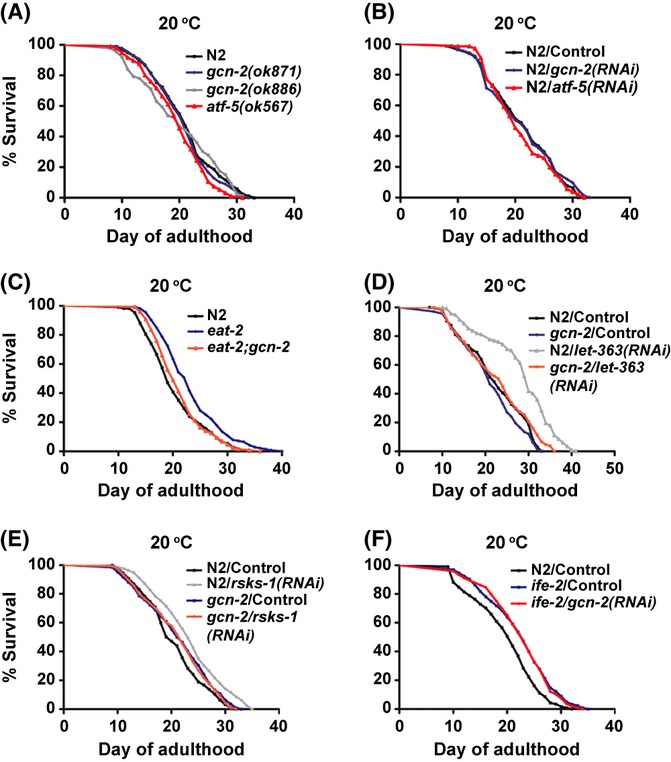
Loss of GCN-2 function affects lifespan only under nutrient stress. Survival curves of (A) N2, *gcn-2,* and *atf-5* mutants fed OP50 bacteria (B) N2 worms treated with *gcn-2(RNAi)* or *atf-5(RNAi)* from L4s (C) *eat-2(ad465)* and *eat-2(ad465);gcn-2(ok871)* fed OP50 bacteria (D) N2 and *gcn-2(ok871)* treated with *let-363(RNAi)* from their first day of adulthood (E) N2 and *gcn-2(ok871)* treated with *rsks-1(RNAi)* from L4s (F) N2 and *ife-2(ok306)* treated with *gcn-2(RNAi)* from L4s.

The conservation of GCN-2 signaling as a nutrient-sensing pathway in *C. elegans* offers the opportunity to address its impact in the aging process, which is strongly affected by the nutrient status of the organism. We performed phenotypic and lifespan analysis in both *gcn-2* (*ok871* and *ok886*) worms as well as in the *atf-5(ok576)* null mutant (Wormbase WS230). All three mutants behaved indistinguishably from N2 strain under normal conditions in growth, development, movement, fecundity (Fig. S3A), and lifespan (Fig. 4A and Table 1). Similarly, postdevelopmental RNAi of *gcn-2* or *atf-5* had no significant effect on the lifespan of N2 worms (Fig. 4B and Table 2). As GCN2 is activated under amino acid limitation, we monitored the lifespan of N2 and *gcn-2* mutants subjected to *rrt-1(RNAi)* or *krs-1(RNAi),* during adulthood only. In all cases, knockdown of either tRNA synthetase gene remarkably shortened lifespan of worms (Fig. S4A and 1 mm isopropylb-D-thiogalactopyranoside (IPTG) in Table 2; Table S3). However, when we applied weaker RNAi conditions to partially inactivate *rrt-1* or *krs-1* gene, we revealed significantly increased sensitivity of both *gcn-2* mutants, compared to N2 (Fig. S4B and 0.25 mm IPTG in Table 2). Worms deficient in GCN-2 kinase activity had a mean lifespan ∼10–15% shorter than the mean lifespan of wild-type. Surprisingly, *atf-5* mutants were not more sensitive than N2 worms under these conditions (Table S3).

**Table 1:** Lifespan experiments in OP-50 plates*

	Strain	Treatment	Median/Max lifespan (days)†	Mean lifespan ± SEM (days)‡	Number (T/C)§	*P*-value against N2¶	*P*-value against specific strain**
Fig. 4A	N2	20 °C	21/30.5	21.33 ± 0.33	99/6		
	*gcn-2(ok871)*	≫	21/29.4	20.67 ± 0.33	80/6	0.4864	
	*gcn-2(ok886)*	≫	20/30	19.33 ± 0.67	88/3	0.7357	
	*atf-5(ok576)*	≫	20/27.7	19.67 ± 0.33	98/3	0.0347	
Fig. 4C	N2	20 °C	19/31	19.4 ± 0.55	110/2		
	*eat-2(ad465)*	≫	23/35.6	23.25 ± 0.25	160/17	0.0001	
	*eat-2(ad465);gcn-2(ok871)*	≫	20/31.4	20.5 ± 0.87	145/14	0.4172	0.0009

* Data from representative experiments are shown. Data from independent repeats of each longevity assay are shown in Table S3 (Supporting information).

† Max lifespan is the mean of the last 10% surviving worms.

‡ Mean lifespan and standard error of the mean (SEM) of 2–4 plates.

§ Total number (T) of dead and censored (C) worms/censored (C).

¶ *P*-value from log-rank test comparing a mutant strain to N2 wild-type strain (<0.05 is considered statistically significant).

** *P*-value from log-rank test comparing a double mutant to a specific single mutant, for example *eat-2;gcn-2* vs. *eat-2* etc.

**Table 2:** Lifespan experiments in RNAi plates*

	Strain/RNAi	Treatment/ IPTG†	Median/Max lifespan (days)‡	Mean lifespan ± SEM (days)§	Number (T/C)¶	*P*-value against control**	*P*-value against specific control††
Fig. 4B	N2/Control	20 °C/1 mm	21/30	20.83 ± 0.44	83/3		
	N2/gcn-2(RNAi)	≫	21/31.3	20.67 ± 0.33	88/3	0.6489	
	N2/atf-5(RNAi)	≫	20/29	19.5 ± 0.29	70/5	0.5680	
Fig. S4 A	N2/Control	20 °C/1 mm	21/30.1	21 ± 0.58	124/5		
	N2/rrt-1(RNAi)	≫	10/16.1	10 ± 0.58	119/10	<0.0001	
	N2/krs-1(RNAi)	≫	8/11.5	7.83 ± 0.16	124/6	<0.0001	
Fig. S4 A	N2/Control	20 °C/1 mm	22/30.4	22 ± 1	118/9		
	N2/rrt-1(RNAi)	≫	13/20.6	13 ± 0.57	125/1	<0.0001	
	*gcn-2(ok871)*/Control	≫	21/30.4	22.17 ± 1.69	117/5		0.0978
	*gcn-2(ok871)*/rrt-1(RNAi)	≫	13/19.1	12.67 ± 0.67	108/6	<0.0001	0.4078
Fig. S4 B	N2/Control	20 °C/0.25 mm	22/30.6	22.50 ± 0.29	80/5		
	N2/rrt-1(RNAi)	≫	19/28.3	18.83 ± 0.44	140/5	<0.0001	
	*gcn-2(ok871)*/Control	≫	22/30.7	21.67 ± 0.67	76/8		0.6962
	*gcn-2(ok871)*/rrt-1(RNAi)	≫	16/27.2	16.33 ± 0.33	138/2	<0.0001	<0.0001
Fig. S4 B	N2/Control	20 °C/0.25 mm	20/28.6	19.67 ± 0.67	91/5		
	N2/rrt-1(RNAi)	≫	17/25.8	16.33 ± 0.33	185/8	<0.0001	
	*gcn-2(ok880)*/Control	≫	19/26	18.67 ± 0.33	92/2		0.1081
	*gcn-2(ok880)*/rrt-1(RNAi)	≫	15/22.9	14.83 ± 0.16	177/6	<0.0001	<0.0001
Fig. S4 B	N2/Control	20 °C/0.25 mm	21/28.6	21.33 ± 0.67	82/12		
	N2/krs-1(RNAi)	≫	12/14.4	12 ± 0.0	96/9	<0.0001	
	*gcn-2(ok871)*/Control	≫	22/27.9	22 ± 1	83/13		0.9383
	*gcn-2(ok871)*/krs-1(RNAi)	≫	11/12.3	11 ± 0.0	79/9	<0.0001	<0.0001
Fig. 4D	N2/Control	20 °C/0.25 mm	22/31.7	21.5 ± 0.5	66/2		
	N2/*let-363(RNAi)*	≫	30/39	29.63 ± 1.14	120/3	<0.0001	
	*gcn-2(ok871)*/Control	≫	21/31.2	21.25 ± 0.75	69/2		0.2995
	*gcn-2(ok871)*/*let-363(RNAi)*	≫	24/34.4	24 ± 1.08	113/4	0.0279	
	*atf-5(ok576)*/Control	≫	21/30.4	21.5 ± 0.5	97/1		0.0456
	*atf-5(ok576)*/*let-363(RNAi)*	≫	27/35.3	27.13 ± 0.77	111/3	<0.0001	
Fig. 4F	N2/Control	20 °C/1 mm	21/29.2	21 ± 0.58	119/2		
	*ife-2(ok306)*/Control	≫	23/32.4	23 ± 0.41	133/7		0.0002
	*ife-2(ok306)*/*gcn-2(RNAi)*	≫	23/31.5	23.25 ± 0.25	124/5	0.1072	
	*ife-2(ok306)*/*atf-5(RNAi)*	≫	23/30.3	22.83 ± 0.44	123/4	0.1696	
Fig. 4E	N2/Control	20 °C/1 mm	20/30	19.75 ± 0.48	112/12		
	N2/*rsks-1(RNAi)*	≫	23/34.1	23.25 ± 0.75	135/5	<0.0001	
	*gcn-2(ok871)*/Control	≫	22/30.8	21.88 ± 0.87	131/18		0.1485
	*gcn-2(ok871)*/*rsks-1(RNAi)*	≫	22/30.2	22.13 ± 0.72	130/8	0.6481	
	*atf-5(ok576)*/Control	≫	19/28	19 ± 0.41	101/15		0.0495
	*atf-5(ok576)*/*rsks-1(RNAi)*	≫	20/30.7	20.75 ± 0.75	134/18	0.0162	

IPTG, isopropylb-D-thiogalactopyranoside.

* Data from representative experiments are shown. Data from independent repeats of each longevity assay are shown in Table S3 (Supporting information).

† IPTG final concentration in bacterial cultures.

‡ Max lifespan is the mean of the last 10% surviving worms.

§ Mean lifespan and standard error of the mean (SEM) of 2–4 plates.

¶ Total number (T) of dead and censored (C) worms/censored (C).

** *P*-value from log-rank test comparing a RNAi-treated strain to isogenic Control strain. *P* < 0.05 is considered statistically significant.

†† *P*-value from log-rank test comparing a mutant strain to the equal treated N2 population, for example *gcn-2/Control* vs. N2*/Control or gcn-2/RNAi* vs. N2*/RNAi*.

Dietary restriction slows aging across animal species and, in *C. elegans*, a well-studied feeding defective mutant that has decreased food uptake and increased lifespan is the *eat-2(ad465)* (Avery, 1993; Lakowski & Hekimi, 1998). We examined the effect of *gcn-2* deletion in the lifespan of *eat-2* worms by creating the double mutant *eat-2(ad465);gcn-2(ok871)*. Loss of GCN-2 activity suppressed the longevity of *eat-2* worms, making them live as wild-type (Fig. 4C and Table 1). This is not due to higher pumping rate of *eat-2;gcn-2*, compared to *eat-2* (data not shown) and *eat-2;gcn-2* worms had similar reduced fecundity as *eat-2* worms (Fig. S3B). In addition, the double mutant exhibited a further delay in development than the single *eat-2* mutant (at 90 h posthatching, ∼30% of *eat-2;gcn-2* animals became adults vs. ∼90% in *eat-2* population), suggesting that *gcn-2* activity is required for both normal growth and longevity of *eat-2* worms.

Several studies in diverse species have linked the DR effect on longevity with the inhibition of the TOR kinase pathway (Kapahi *et al*., 2004; Kaeberlein *et al*., 2005; Hansen *et al*., 2008). We examined whether loss of GCN-2 signaling affects the long life of worms with reduced TOR (LET-363) kinase activity. By subjecting N2, *gcn-2,* and *atf-5* worms in *let-363(RNAi)* from their first day of adulthood, we observed that TOR inhibition failed to increase lifespan in *gcn-2* mutants (Fig. 4D and Table 2). We verified by quantitative reverse transcription PCR (qRT–PCR) that *gcn-2* mutants were responsive to feeding RNAi at the same level as N2 (Fig. S5). Two downstream effectors of the TOR pathway are the *rsks-1/*S6 kinase and *ife-2/*eIF4E translation initiation factor, which when inactivated extends worm’s lifespan (Hansen *et al*., 2007; Pan *et al*., 2007; Syntichaki *et al*., 2007). We found that inactivation of *rsks-1* failed to increase the lifespan in *gcn-2* mutants (Fig. 4E and Table 2). However, inactivation of *gcn-2* gene in *ife-2(ok306)* worms did not alter their long life (Fig. 4F and Table 2). Finally, loss of *atf-5* did not alter the longevity induced by inactivation of TOR and its effectors (Table 2), suggesting that loss of ATF-5, in contrast to GCN-2, is not sufficient to alter lifespan of nutrient-sensitized worms.

### General control nonderepressible-2 regulates the activation of PHA-4 in response to TOR disruption

**Figure 5:**
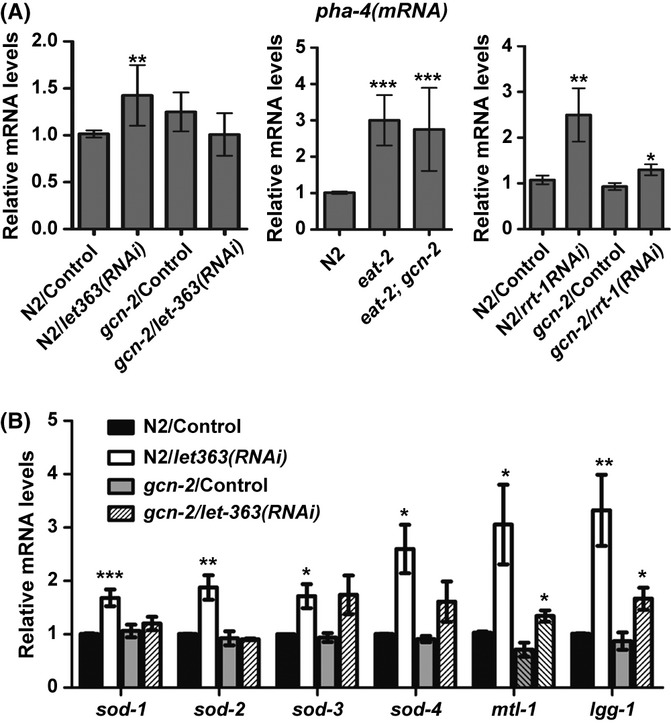
General control nonderepressible-2 influences the induction of *pha-4* and its downstream targets in response to TOR inactivation. qRT–PCR of *pha-4(mRNA)* in (A) 1-day adults of N2 and *gcn-2(ok871)* treated with *let-363(RNAi)* from L3 stage; N2, *eat-2,* and *eat-2;gcn-2* young adults raised on OP-50 bacteria at 20 °C; and in the F1 progenies (L2–L3 stage) of N2 and *gcn-2(ok871)* treated with *rrt-1(RNAi*) (B) qRT–PCR of *sod-1*, *sod-2*, *sod-3*, *sod-4*, *mtl-1,* and *lgg-1* on 1-day adults of N2 and *gcn-2(ok871)* treated with *let-363(RNAi)* from L3 stage. Quantification of each mRNA level, relative to *ama-1* mRNA and the mean ± SD of biological triplicates are shown. The asterisks represent statistical significant difference from N2/Control or *gcn-2*/Control (*P < 0.05, ** *P* < 0.01, ***P < 0.001 in unpaired *t*-test).

In *C. elegans*, the transcription factor PHA-4/FoxA is selectively required for lifespan extension by DR or reduced TOR signaling (Panowski *et al*., 2007; Steffen *et al*., 2008). It was also shown that inactivation of *let-363* and *rsks-1*, but not *ife-2*, promotes PHA-4 activity to increase lifespan, indicating that the TOR and *rsks-1*/S6K signaling antagonizes PHA-4 (Steffen *et al*., 2008). The expression levels of *pha-4* are increased in TOR-deficient and *eat-2(ad1116)* animals (Panowski *et al*., 2007; Lapierre *et al*., 2011). We observed similar transcriptional induction of *pha-4* in *eat-2(ad465)*, N2/*let-363(RNAi),* and N2/*rrt-1(RNAi*) worms, but this induction was impaired in the absence of GCN-2, at least for the RNAi-treated animals (Fig. 5A). Thus, GCN-2 signaling positively regulates *pha-4* transcript levels in response to reduced TOR pathway.

It has been suggested that increased expression of *pha-4* during DR or TOR inhibition facilitates its binding to DR-specific genes, in an analogous manner to its expression and binding specificity during embryogenesis (Panowski *et al*., 2007). Such PHA-4 targets are genes mostly involved in metabolic processes and defense responses (Panowski *et al*., 2007; Zhong *et al*., 2010). The superoxide dismutase (*sod*) gene family, responsible for scavenging ROS, includes members that are differentially regulated by PHA-4 and DAF-16/FoxO transcription factors in response to DR or reduced insulin/IGF-1 signaling (ISS), respectively. PHA-4 regulates the expression of *sod-1*, *sod-2*, *sod-4,* and *sod-5*, but not *sod-3*, in *eat-2* mutants, while DAF-16 regulates the expression of *sod-1*, *sod-3,* and *sod-5* in ISS mutants (Panowski *et al*., 2007). By measuring the mRNA levels of *sod* genes in *let-363(RNAi)*-fed worms, we found a clear induction of PHA-4-regulated *sod-1*, *sod-2,* and *sod-4* (*sod-5* was not tested) (Fig. 5B), similarly to *eat-2* mutants. Moreover, this induction was GCN-2-dependent (Fig. 5B). Curiously, we observed an induction of DAF-16-regulated *sod-3* in *let-363(RNAi)*-fed worms, which was totally GCN-2-independent (Fig. 5B). Although TOR disruption extends lifespan independently of DAF-16, the induction of *sod-3* in TOR-deficient worms might be due to reduced ISS and activation of DAF-16 or alternatively, a mild stress response to elevated mitochondrial ROS under these conditions (Ristow & Zarse, 2010). Evidence against the first hypothesis comes from the lower induction of *sod-3* here than in response to lower ISS and that the expression of another DAF-16 target, *hsp-16.2*, did not change in *let-363(RNAi)*-treated worms (data not shown).

We also tested the expression of *mtl-1* gene, encoding for a metallothionein involved in detoxification/stress adaptation. This is a DAF-16 target under reduced ISS, but is a candidate PHA-4 target in starved L1s (Zhong *et al*., 2010). We observed an upregulation of *mtl-1* transcript in *let-363(RNAi)*-treated animals, which was partially GCN-2-dependent (Fig. 5B). PHA-4 is also directly involved in the induction of autophagy-related genes, and autophagy is required for lifespan extension in response to DR and TOR disruption (Jia & Levine, 2007; Hansen *et al*., 2008; Zhong *et al*., 2010; Lapierre *et al*., 2011). Again, we measured less induction of the autophagic gene *lgg-1* in *let-363(RNAi)*-fed worms when GCN-2 activity was missing, compared to controls (Fig. 5B). Similarly, the induction of *mtl-1* and *lgg-1* was diminished in *eat-2;gcn-2* compared to *eat-2* worms (Fig. S6). Taken together, loss of GCN-2 impairs the induction of *pha-4* and specific downstream targets, under conditions of DR or TOR inactivation.

### General control nonderepressible-2 signaling has a protective role against a multitude of stresses

**Figure 6:**
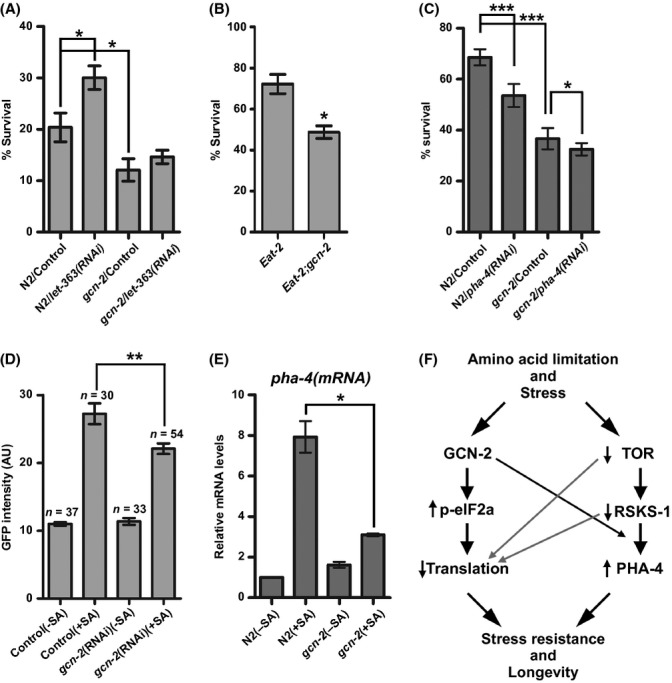
GCN-2 affects *pha-4* induction and survival of TOR-deficient and *eat-2* worms under oxidative stress. Survival to sodium arsenite (SA) of 1-day adults of (A) N2 and *gcn-2(ok871)* fed with *let-363(RNAi)* from L3 stage (B) *eat-2(ad465)* and *eat-2(ad465);gcn-2(ok871)* (C) N2 and *gcn-2(ok871)* fed with *pha-4(RNAi)* from eggs (D) Quantification in arbitrary units (AU) of GFP signal in the intestine of 1-day adults expressing a membrane-bound GFP under the *pha-4* promoter, fed either Control or *gcn-2(RNAi)* expressing bacteria and treated or not with SA (15 mm for 3 h before observation). Fluorescence intensity was measured from several confocal images using ImageJ. The total number (*n*) of areas counted and the mean ± SD are shown (E) qRT–PCR of *pha-4(mRNA)* in 1-day N2 or *gcn-2* worms treated or not with SA (15 mm for 1.5 h). Quantification of each mRNA level, relative to *ama-1* mRNA, and the mean ± SD of biological triplicates are shown (*P < 0.05, **P < 0.01, ***P < 0.001 in unpaired *t*-test) (F) A model illustrating the function of GCN-2 in response to nutrient and other stresses.

Inactivation of various components of the TOR pathway in yeast and *C. elegans* (Powers *et al*., 2006; Hansen *et al*., 2007; Pan *et al*., 2007; Syntichaki *et al*., 2007) leads to increased stress resistance, but the underlying mechanisms remain elusive. The transcriptional activity of PHA-4 under DR or reduced TOR should be part of an adaptive mechanism of cells to cope with environmental stresses and live longer. Based on our findings that GCN-2 activity positively regulates expression of PHA-4 and some of its targets, which are genes involved in stress defense, we assessed sensitivity of TOR-deficient worms to oxidative stress, in the presence or absence of GCN-2. Whereas *let-363(RNAi)*-treated animals were more resistant to sodium arsenite than untreated controls, the deletion of *gcn-2* partially suppressed this resistance (Fig. 6A). Likewise, *eat-2* worms were more resistant to sodium arsenite than the *eat-2;gcn-2* (Fig. 6B). We also observed that knockdown of *pha-4* decreased the percentage of N2 that survived this stress but had smaller effect on *gcn-2* worms, which were more sensitive than N2 (Fig. 6C). As our data suggest that oxidative stress can induce *pha-4* in a GCN-2-dependent manner, we confirmed this by quantification of the fluorescent signal of *a pha-4* promoter-driven GFP reporter in N2 and *gcn-2(RNAi)* worms, under sodium arsenite (Figs 6D and S7). This was also verified by measuring the mRNA levels of the endogenous *pha-4* gene in N2 and *gcn-2*, treated with sodium arsenite (Fig. 6E). Finally, we observed increased sensitivity of *gcn-2* worms after heat shock or UV irradiation, compared to N2 (Fig. S8). Combined these results suggest a broad role of GCN-2 on stress response and survival, through its function on translation regulation and/or transcriptional induction of specific programs that adapt cells to each stress (Fig. 6F).

## Discussion

Aging is a complex biological process critically influenced by endogenous or exogenous signals that affect basic mechanisms and pathways, related to metabolism and stress response. Reduced nutrient signals can effectively alter the lifespan in many organisms, and nutrient-sensing pathways have acquired central role in the longevity determination. One such is the TOR kinase pathway that regulates both anabolic and catabolic processes, important for stress management and long-term survival. Another nutrient-sensing pathway is that of GCN2 kinase, which phosphorylates eIF2α translation factor in response to nutrient or other stresses. However, the impact of GCN2 function and its possible connection to TOR pathway in lifespan determination have not been investigated. *C. elegans* is a primary model organism for aging studies and has profoundly contributed to the determination of genetic and environmental factors that affect aging in organismal level.

We established the conserved role of *C. elegans* GCN-2 as an eIF2α kinase under nutrient stress and showed that the mRNA of *atf-5*, encoding a worm homolog of yeast GCN4 and mammalian ATF4 transcription factors, is under translational control by GCN-2. In normal culture conditions, deletion of *gcn-2* was dispensable for growth, fertility, and lifespan of worms. However, under amino acid limitation, loss of *gcn-2* shortened lifespan, supporting its function under nutrient deprivation. Furthermore, *gcn-2* deletion decreased the long lifespan of nutrient-responsive worms, such as *eat-2* mutants, a genetic model of DR (Lakowski & Hekimi, 1998), or RNAi-treated worms for *let-363/*TOR, and its downstream target *rsks-1/*S6K. We have revealed a novel role of GCN-2 in modulating *pha-4/*FoxA expression under conditions of TOR inactivation or amino acid deprivation. PHA-4/FoxA transcription factor is a master regulator of organ development, but is also required for survival of larvae under starvation and DR-induced longevity in adults (Hansen *et al*., 2007; Panowski *et al*., 2007; Sheaffer *et al*., 2008; Zhong *et al*., 2010). Thousands of genes are candidate PHA-4 targets, with diverse roles in many biological processes and preferentially bound in specific conditions (Zhong *et al*., 2010).

Under DR, PHA-4 activity regulates the expression of many autophagy-related genes or certain *sod* genes, encoding superoxide dismutase isoforms (Morck & Pilon, 2006; Panowski *et al*., 2007; Hansen *et al*., 2008). Here we showed that the induction of such stress-responsive genes in *let-363(RNAi)*-treated worms was dependent on GCN-2, consistent with the lower induction of *pha-4* in *gcn-2* mutants, relative to N2. Accordingly, *gcn-2* deletion rendered wild-type, *eat-2,* or TOR-deficient worms more sensitive to sodium arsenite-induced oxidative stress, compared to the GCN-2 proficient animals. Also *gcn-2* worms were more susceptible to other stresses, such as heat shock and UV irradiation. Lately, it was shown that activation of GCN-2 signaling protects cells during mitochondrial and hypertonic stress (Baker *et al*., 2012; Lee & Strange, 2012). A common cellular response to stress is the inhibition of global translation and induction of specific translational/transcriptional programs to maintain their intracellular homeostasis. GCN-2 has a central role in this response and participates in stress management by activating key transcription factors, such as ATF4 and NF-kB in mammals (Wek *et al*., 2006). This stress-induced reprogramming would also determine lifespan. A role of the yeast GCN4 in the lifespan extension by mutations in TOR, S6K, and 60S subunits has been reported (Steffen *et al*., 2008). Although GCN-2 increases *atf-5* synthesis under amino acid limitation, loss of *atf-5* did not recapitulate the effect of *gcn-2* deletion in stress survival and lifespan. We speculate that ATF-5 has either more specific roles not related to the longevity mechanisms in worms or redundant function(s) with other transcription factors (e.g., PHA-4), so worms can compensate for its loss.

Overall, we demonstrated the central role of worm GCN-2 in stress response and revealed that the PHA-4 transcription factor is part of the GCN-2 signaling in response to nutrient and oxidative stress. Although longevity is not always linked to increased stress resistance and overexpression of antioxidants genes does not necessary lead to longevity, such an association could rely on specific genetic or environmental backgrounds. Studies in yeast, worms, flies, and mice suggest that an induction of oxidative stress and mitochondrial respiration may be a mechanism of DR-induced longevity (Ristow & Zarse, 2010; Pan *et al*., 2011). In *Drosophila*, SOD1 is required for lifespan extension by protein restriction only when sugar level is high (Sun *et al*., 2012). Conversely, genetic data in *C. elegans* support that oxidative stress is uncoupled from aging, and deletions of individual or combinations of antioxidant genes did not reduce the lifespan of N2, *eat-2,* or ISS mutants (Van Raamsdonk & Hekimi, 2010). This might be due to their small contribution on the whole adaptive response that determines longevity. This adaptive response should involve a coordinated function of several genes and pathways, regulated by transcription factors with a broad cellular role.

The function of PHA-4 in promoting survival of L1 larvae under starvation is dictated by the expression of several targets, mostly related to stress defense and metabolic processes (Zhong *et al*., 2010). It is likely that the function of PHA-4 in the modulation of adult lifespan extension under DR entails similar targets and cellular processes. Increase in antioxidant defense and catabolic processes such as autophagy protect cells from the accumulation of damaged proteins or organelles. On the other hand, metabolic alterations induced by mitochondrial function or regulators of fatty acid metabolism could significantly modulate longevity. In yeast, activation of Rim15 kinase by nutrient depletion positively regulates the stress-responsive transcription factors Msn2/4 and Gis1 to protect cells and increase lifespan under DR and TOR inhibition (Powers *et al*., 2006; Wei *et al*., 2009). This is accomplished through the induction of stress defense genes and by switching metabolism from respiration to glycolysis and glycerol synthesis, generating a DR-like environment that enhances stress survival and lifespan (Wei *et al*., 2009). Our work provides the first genetic evidence that GCN-2 activity can influence the outcome of DR effect on lifespan by modulating the TOR/S6K signaling and its downstream transcription factor PHA-4. GCN-2 signaling positively regulates the induction of PHA-4 under nutrient or oxidative stress, by a yet unidentified mechanism. The next challenge will be to identify the downstream targets of TOR pathway that are controlled by GCN-2 and contribute to stress resistance and longevity.

## Experimental procedures

### *Caenorhabditis elegans* strains and culture

Standard methods of culturing and handling worms were used. Worms were raised on NGM plates seeded with *E. coli* OP50 as food. For tunicamycin treatments, L4 larvae were transferred on NGM plates with tunicamycin (5 μg/mL) for 24 h. See Table S1 (Supporting information) for all strains used in this study. Wild-type Bristol N2 and single mutant strains were provided by the Caenorhabditis Genetics Center (CGC, University of Minnesota, Minneapolis, MN, USA). The *gcn-2(ok871)*, *gcn-2(ok886),* and *atf-5(ok576)* were outcrossed four times with the N2, and the relevant mutations were tracked in F2 progeny by PCR. For genotyping the mutants, primers G1/G2 and G1/G3 (Table S2) were used for both *gcn-2* alleles vs. N2, and for *atf-5(ok576),* the set of primers A1/A2 (Table S2). The double mutant *eat-2(ad465);gcn-2(ok871)* was made by crossing the desired strains and selecting F2 progeny by PCR for carrying both mutations. To track the ad465 point mutation in *eat-2,* a PCR fragment using primers E1/E2 (Table S2) was digested with the restriction enzymes BamHI-SfcI. Transgenic animals were generated by microinjection of plasmid DNAs into the gonad of young adult N2 worms, using *rol-6(su1006)* as cotransformation marker. BRF144 was obtained by genetic cross of BRF140 with BRF162 males (Table S1).

### Constructs

RNAi plasmids were constructed by inserting gene-specific PCR product, amplified from genomic DNA using the relevant primers (Table S2), into the RNAi feeding vector pL4440 (Andy Fire Kit 1999, Addgene plasmid 1654, Cambridge, MA, USA). For *eIF2α(RNAi)* plasmid, a KpnI digest fragment from pHSG399-eIF2α plasmid (provided by Dr. Shin Takagi (Nukazuka *et al*., 2008)) was subcloned to pL4440. RNAi clone for *let-363* and *rsks-1* was previously described (Syntichaki *et al*., 2007). For the intact *atf-5::gfp* transgene, a genomic PCR fragment with the A1/A4 primers (Table S2) containing the 1612 bp sequence upstream of the *atf-5* coding region, and the 1413 bp *atf-5* coding region (including uORFs) was cloned into the pPD95.77 vector (Andy Fire Kit 1995, Addgene plasmid 1494, Cambridge, MA, USA) in fusion with *gfp* at the C’-terminal. For the uORF-less *atf-5::gfp*, we first cloned the promoter region of *atf-5*, amplified with the set of primers A1/A2 (Table S2), into the pPD95.77 vector, and in this plasmid (P_*atf-5*_::GFP), we added the coding region of *atf-5* without uORFs, amplified with the primers A5/A4 (Table S2).

### Western blot analysis

Worms of each strain, grown on OP50 or RNAi plates at 20 °C, were collected in M9 buffer when just reaching adulthood. After 2–3 washes to remove bacteria, they were frozen in ethanol dry ice and, before loading onto SDS-PAGE, worm pellets were boiled in 50 μL 2X SDS-sample buffer for 10 min. Primary antibodies were a polyclonal antibody raised against worm eIF2α, a kind gift from Dr. Shin Takagi (Nukazuka *et al*., 2008), for total (T)-eIF2α and an anti-phospho-eIF2α antibody (Santa Cruz Biotechnology, sc-101670) for phosphorylated (P)-eIF2α levels. A secondary anti-rabbit IgG antibody (HRP) was used for immunoblot signal detection with ECL (Thermo Fisher Scientific Inc., Waltham, MA, USA). Quantification of immunoblot signals was performed using ImageJ software (ImageJ, U. S. National Institutes of Health, Bethesda, MD, USA). Ratio of P- to T-eIF2α levels was measured in two independent experiments.

### RNA interference

The RNAi clones, expressing dsRNA from the indicated genes in HT115(DE3) *E. coli* bacteria, were grown with ampicillin (50 μg/mL) and tetracycline (10 μg/mL) in LB medium. On the following days, fresh cultures with ampicillin were induced with 0.25 or 1 mm isopropylb-D-thiogalactopyranoside (IPTG) and seeded on RNAi plates. Bacteria carrying the empty vector (pL4440) and treated likewise were used as control cultures.

### Microscopy

The expression pattern of transgenic worms was monitored by mounting sodium azide- or levamizole-treated animals on 2% agarose pads, on glass microscope slides. Animals were imaged either under fluorescence microscope or using a Leica TCS SP5 confocal imaging system. Images shown from confocal are 2D maximal projections of z-stacks.

### RNA isolation and quantitative reverse transcription PCR

Total RNA was prepared from frozen worm pellets, of the indicated genetic backgrounds and developmental stages, using a NucleoSpin RNA XS kit (Macherey–Nagel, Dueren, Germany) and measured by Quant-iT RNA assay kit (Invitrogen, Molecular Probes, Eugene, OR, USA). Total RNA was reverse transcribed with iScript™ cDNA synthesis kit (Biorad, Hercules, CA, USA), and quantitative PCR was performed using the SsoFast™ EvaGreen supermix (BioRad) in the MJ MiniOpticon system (BioRad). The relative amounts of mRNA were determined using the comparative Ct method for quantification, and the gene expression data are presented as the fold change in each strain relative to N2. qRT–PCR was performed in at least two independent samples in triplicates, and each sample was independently normalized to endogenous reference *ama-1*. The mean ± the standard deviation (SD) of at least two independent experiments is presented. The sequence of primers used for qRT–PCR is available upon request.

### Lifespan assays

Lifespan assays were conducted as described previously (Syntichaki *et al*., 2007). Briefly, L4 larvae of each strain were transferred to NGM plates or RNAi plates, at the assay temperature. Animals were transferred every two days to fresh plates and were daily scored for surviving worms. Animals that failed to respond to stimulation by touch were referred as dead, whereas that bagged, exploded, or crawled off the plates were referred as censored in the analysis. Day 0 of adulthood was defined the day that the L4s were transferred to plates. Lifespan and statistical analysis were performed using GraphPad Prism, version 5 (GraphPad Software, San Diego, CA, USA). Each population is compared with the appropriate control population using the log-rank test.

### Fertility assay

Worms of each genotype were grown at 20 °C, and 5–10 L4 hermaphrodites were placed on individual NGM plates to lay eggs. Animals were transferred daily to fresh plates until egg-laying ceased and the hatched progeny were counted in each plate. The total number of progeny per worm (brood size) was counted for each genotype, and the average brood size (mean ± SD) of each strain was plotted. Unpaired *t-*test was used to calculate *P*-values in GraphPad Prism 5.

### Stress resistance assays

For heat-shock assays, 1-day-old worms were shifted at 35 °C for 6 h. After one night of recovery at 20 °C, the percentage of worms surviving was determined. For UV resistance assays, 5-day adults were irradiated to NGM plates without bacteria at 0.2J/cm^2^ and then were transferred to NGM plates with food at 20 °C. Three days later, the percentage of worms surviving was determined. For oxidative stress, 1-day adults of each strain were transferred on NGM plates with 3 mm (for *eat-2* and *eat-2;gcn-2*) or 5 mm (for N2 and *gcn-2*) sodium arsenite, and the percentage of worms surviving was determined after 20 h or 48–58 h at 20 °C, respectively. The average (mean ± SD) of at least three independent experiments with ∼100 individual for each strain per experiment was plotted. Unpaired *t*-test was used to calculate *P*-values in GraphPad Prism 5.

## Supporting Information

**Fig. S1** Inactivation of leucyl-tRNA synthetase *lrs-1* induces phospho-eIF2α levels in a *gcn-2*-dependent manner. **Fig. S2** Induction of *atf-5::gfp* transgene by *eIF2α(RNAi)* or tunicamycin does not require GCN-2 activity. **Fig S3** *gcn-2* deletion does not alter fertility in wild-type or *eat-2* mutants. **Fig. S4** Loss of GCN-2 activity sensitizes animals to amino acid limitation. **Fig. S5.** RNAi efficiency is not affected in *gcn-2* mutant worms. **Fig. S6** GCN-2 regulates the induction of PHA-4 target genes in *eat-2* mutants. **Fig. S7** Inactivation of *gcn-2* affects the induction of a P_*pha-4*_*::gfp* reporter under oxidative stress. **Fig. S8** Loss of *gcn-2* but not *atf-5* increases the stress sensitivity of worms. **Table S1** List of the strains used in this study. **Table S2** List of primers used for plasmid construction in this study. **Table S3** Summary of data from independent repeats of lifespan experiments.
