# Supplementary material for: The general control nonderepressible-2 kinase mediates stress response and longevity induced by target of rapamycin inactivation in Caenorhabditis elegans
Source: Aging Cell. 2013 Jun 28;12(5):742–51. doi: 10.1111/acel.12101 (PMC4225475; doi:10.1111/acel.12101)
Supplement: Supplementary file 1 — Fig. S1 Inactivation of leucyl-tRNA synthetase lrs-1 induces phospho-eIF2α levels in a gcn-2-dependent manner. Fig. S2 Induction of atf-5::gfp transgene by eIF2α(RNAi) or tunicamycin does not require GCN-2 activity. Fig S3 gcn-2 deletion does not alter fertility in wild-type or eat-2 mutants. Fig. S4 Loss of GCN-2 activity sensitizes animals to amino acid limitation. Fig. S5. RNAi efficiency is not affected in gcn-2 mutant worms. Fig. S6 GCN-2 regulates the induction of PHA-4 target genes in eat-2 mutants. Fig. S7 Inactivation of gcn-2 affects the induction of a Ppha-4::gfp reporter under oxidative stress. Fig. S8 Loss of gcn-2 but not atf-5 increases the stress sensitivity of worms. Table S1 List of the strains used in this study. Table S2 List of primers used for plasmid construction in this study. Table S3 Summary of data from independent repeats of lifespan experiments. [file acel0012-0742-sd1.pdf]

Supporting Information

Fig. S1

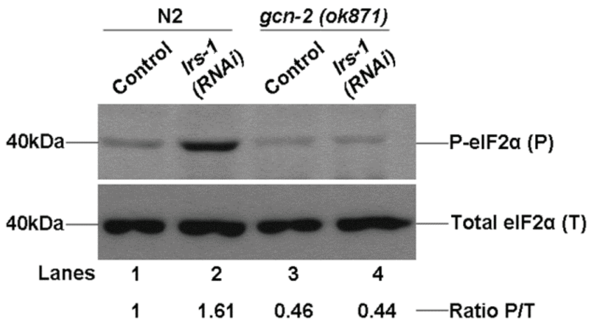

Fig. S1: Inactivation of leucyl-tRNA synthetase *lrs-1* induces phospho-eIF2α levels in a *gcn-2*-dependent manner. Western blot analysis showing the levels of phosphorylated eIF2α (P-eIF2α), normalized by the total amount of eIF2α, in whole extracts of 1-day N2 and *gcn-2(ok871)* worms, fed RNAi bacteria harboring the empty vector (Control) or expressing dsRNA for *lrs-1*.

Fig. S2

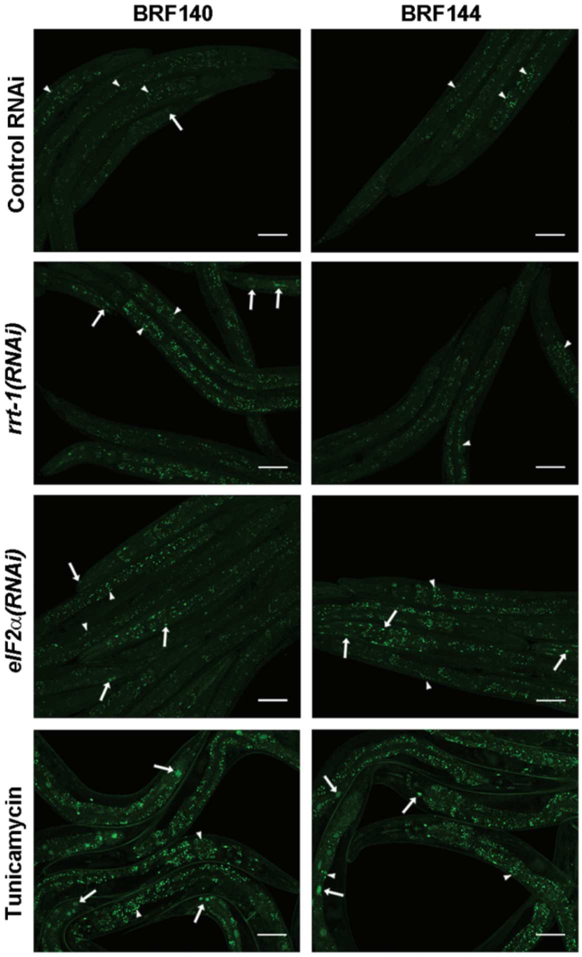

Fig. S2: Induction of *atf-5::gfp* transgene by *eIF2α(RNAi)* or tunicamycin does not require GCN-2 activity. Confocal images of adult transgenic worms BRF140 (N2Ex[ATF-5::GFP; pRF4]) and BRF144 (*gcn-2(ok871)*Ex[ATF-5::GFP; pRF4]) that were fed Control, *rrt-1(RNAi)* or *eIF2α(RNAi)* bacteria, or were treated with tunicamycin (5μg/ml for 24h). White arrows indicate fluorescent nuclei and white arrowheads show regions of autofluorescence. All images were taken at 20x magnification (scale bar: 50μm).

Fig. S3

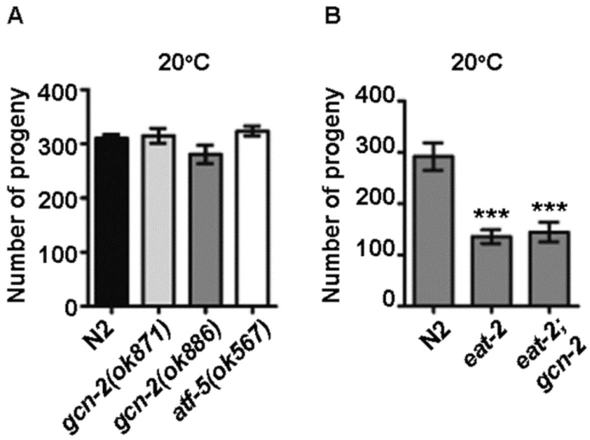

Fig. S3: *gcn-2* deletion does not alter fertility in wild-type or *eat-2* mutants. Brood size (mean ± SD) of 5-10 individuals of the indicated strains at 20°C: **(A)** N2, *gcn-2(ok871)*, *gcn-2(ok886)* and *atf-5(ok567)*. **(B)** N2, *eat-2* and *eat-2;gcn-2* mutant animals.The asterisks represent statistical significant difference from N2 (***p<0.001 in unpaired t-test).

Fig. S4

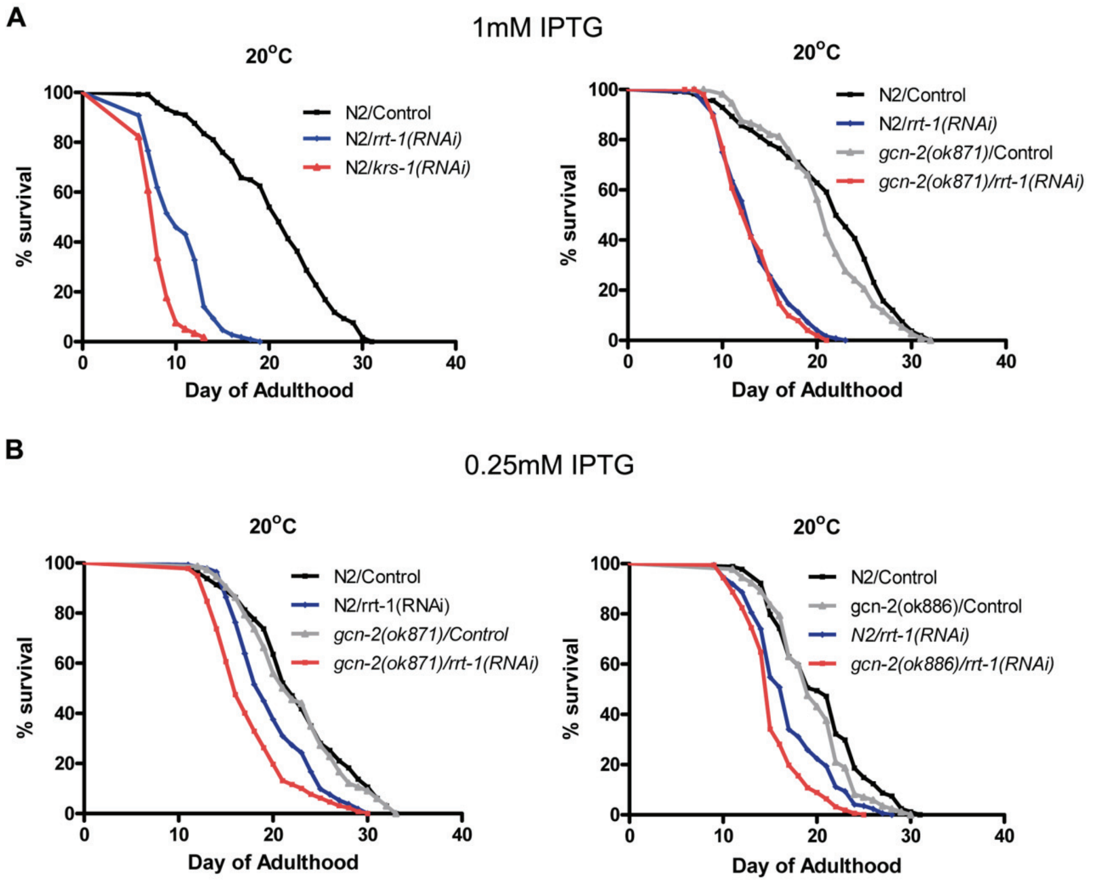

Fig. S4: Loss of GCN-2 sensitizes animals to amino acid limitation. **(A)** Survival curves of N2 and *gcn-2* mutant worms subjected to strong RNAi conditions (1mM IPTG) to inactivate *rrt-1* or *krs-1*, compared to empty vector (Control), at 20°C. **(B)** Survival curves of N2 and *gcn-2* mutant worms subjected to weak RNAi conditions (0.25mM IPTG) to inactivate *rrt-1* gene, compared to empty vector (Control), at 20°C. See Table 2 for additional data.

Fig. S5

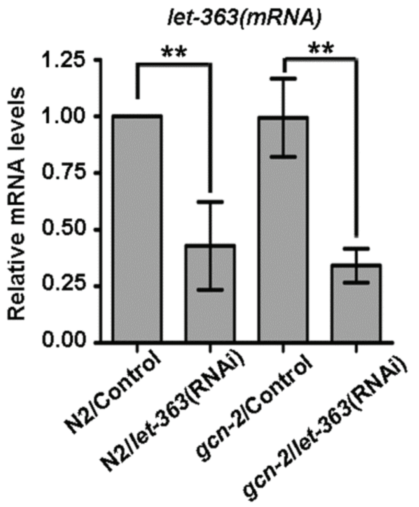

Fig. S5: RNAi efficiency is not affected in *gcn-2* mutant worms. qRT-PCR of *let-363* transcript on N2 and *gcn-2* worms, subjected to Control RNAi or *let-363(RNAi)* expressing bacteria for 4 days. The asterisks represent statistical significant difference (*p<0.05, **p<0.01, ***p<0.001 in unpaired t-test).

Fig. S6

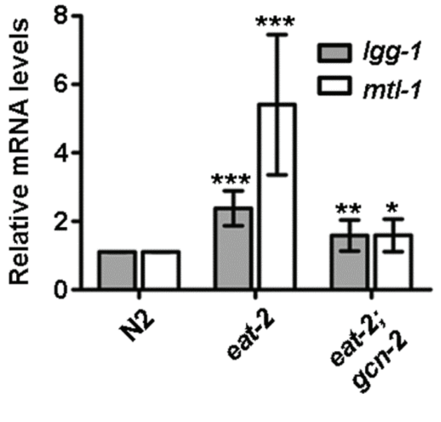

Fig. S6: GCN-2 regulates the induction of PHA-4 target genes in *eat-2* mutants. qRT-PCR of *lgg-1* and *mtl-1* transcript levels in 1-day adults of N2, *eat-2* and *eat-2;gcn-2* raised on OP-50 bacteria at 20°C. The asterisks represent statistical significant difference from N2 (*p<0.05, **p<0.01, ***p<0.001 in unpaired t-test)

Fig. S7

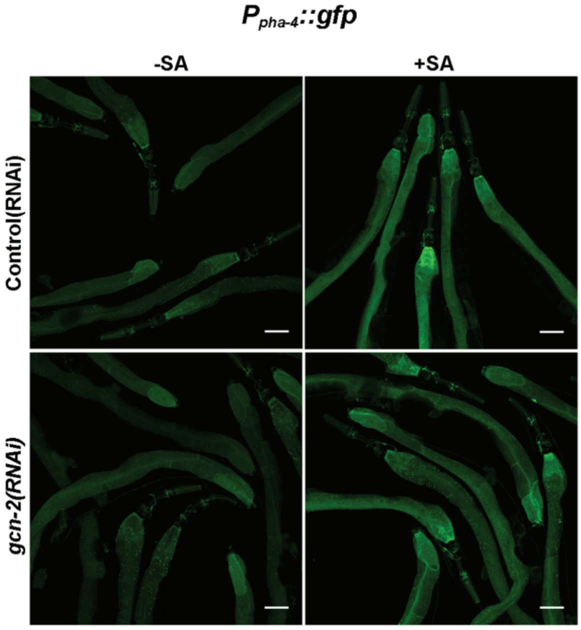

Fig. S7: Inactivation of *gcn-2* affects the induction of a P_{pha-4}::*gfp* reporter under oxidative stress. Confocal images of 1-day adults expressing a membrane-bound GFP under the *pha-4* promoter (SM481strain), fed for two generations either Control RNAi or *gcn-2(RNAi)* expressing bacteria and treated (+SA) or not (-SA) with sodium arsenite (15mM for 3h before observation). All images were taken at 20x magnification under the same microscopy settings (scale bar: 50µm).

Fig.S8

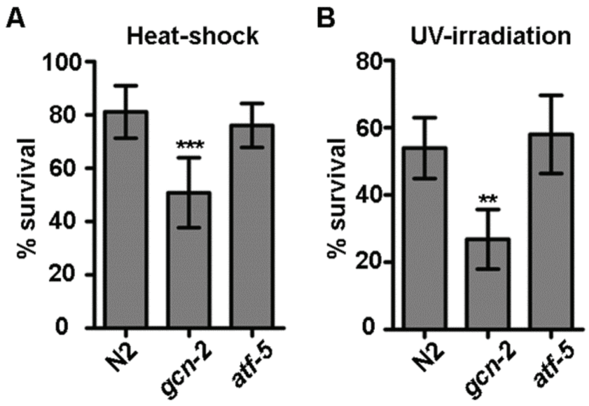

Fig. S8: Loss of *gcn-2* but not *atf-5* increases the stress sensitivity of worms. **(A)** Survival of 1-day old N2, *gcn-2* and *atf-5* adults subjected to heat-shock (35^oC for 6h). **(B)** Survival of 5-day old N2, *gcn-2* and *atf-5* adults after UV-irradiation ($0.2J/cm^2$).

Table S1: List of the strains used in this study

Strain	Genotype	Information	Reference
N2		Wild-type	
BRF162	*gcn-2(ok871 II)*	Backcrossed to N2 4x	This study
BRF163	*gcn-2(ok881 II)*	Backcrossed to N2 4x	This study
BRF172	*atf-5(ok576 X)*	Backcrossed to N2 4x	This study
DA465	*eat-2(ad465 II)*	Point mutation of Y48B6A.4. Backcrossed to N2 1x	CGC
BRF178	*gcn-2(ok871I);eat-2(ad465 II)*	From crossing of BRF162 with DA465	This study
BRF140	N2 Ex[ATF-5::GFP;pRF4 *rol-6(su1006)*]	Translational fusion of the intact gene T04C10.4 under its promoter	This study
BRF144	*gcn-2(ok871)*Ex[ATF-5::GFP;pRF4 *rol-6(su1006)*]	From crossing of BRF140 with BRF162	This study
BRF152	N2 Ex[$_{\text{-uORFs}}$ATF-5::GFP;pRF4 *rol-6(su1006)*]	Translational fusion of the uORF-less gene T04C10.4 under its promoter	This study
SM481	pxIs10[$P_{pha\text{-}4}$::GFP::CAAX ; pRF4 *rol-6(su1006)*]	Membrane-bound GFP under the *pha-4* promoter	CGC
KX15	*ife-2(ok306 X)*	Deletion of R04A9.4 (*ife-2*)	CGC

Table S2: List of primers used in this study

Primer	Sequence (5' to 3')	Used for
GCN-2/1 (G1)	GCGATTGATGTTGTTCCAG	*gcn-2(ok871* and *ok886)* genotyping
GCN-2/2 (G2)	GAGACCACATCCATCGC	>>
GCN-2/3 (G3)	GTGAGTAGACTCGTCCG	>>
ATF-5/1 (A1)	ACATGGCATGCATGATTTATAACGGAAGTTCAG	*atf-5(ok576)* genotyping
ATF-5/2 (A2)	GGTCTAGAAATTTTCAGATGAGATGTTTCTGCG	>>
Eat-2/1 (E1)	GCTAGTCGATTTTCATCATCG	*eat-2(ad465)* genotyping
Eat-2/2 (E2)	GGCTAACCTTCAAATAGCAAAC	>>
GCN-2/4 (G4)	CTACCTACTCTCGAGTTCC	*gcn-2(RNAi)* construct
GCN-2/6 (G6)	AACTGCAGCTCATTGCTTCCAGCG	>>
ATF-5/3 (A3)	AACTGCAGAGTCGTCTCCCTTTCCTC	*atf-5(RNAi)* construct
ATF-5/4 (A4)	CGGGATCCGTCGGTGACAGTTTTCATTC	>>
RRT-1/1 (R1)	GGCAGATCTGGATACTCTGACTACTCAG	*rrt-1(RNAi)* construct
RRT-1/2 (R2)	GGCCTGCAGCAGCTTCACAACATACTCG	>>
KRS-1/1 (K1)	GCGCCATGGCAAGCCAAGAAGGAACAAG	*krs-1(RNAi)* construct
KRS-1/2 (K2)	GGGCTGCAGCGAGTGGTGACATGATTTG	>>
ATF-5/1 (A1)	ACATGGCATGCATGATTTATAACGGAAGTTCAG	$P_{atf\text{-}5}$*::atf-5::gfp* construct
ATF-5/4 (A4)	CGGGATCCGTCGGTGACAGTTTTCATTC	>>
ATF-5/2(A2)	GGTCTAGAAATTTTCAGATGAGATGTTTCTGCG	$P_{atf\text{-}5}$*::gfp* construct
ATF-5/5 (A5)	GGTCTAGAATGGCTTATGTAAATGAACAAAATCC	$_{\text{-}uORF}$*::atf-5::gfp* construct
LRS-1/1 (L1)	GGCAAGCTTCAAGGATGATAAGGGAACTGG	*lrs-1(RNAi)* construct
LRS-1/2 (L2)	GGCCTCGAGCACGAAGCATCTGTCATCTG	>>
PHA-4/1 (P1)	CCCAAGCTTGCGGTCATCGGAAGAAGC	*pha-4(RNAi)* construct
PHA-4/2 (P2)	CCGCTCGAGCTGGTATACTCCGTTGGTG	>>

Table S3: Summary of Data from independent repeats of lifespan assays

	Strain/RNAi	Treatment (Temp/IPTG)	Median/Max Lifespan (days)[b]	Mean lifespan ± s.e.m (days)[c]	Number (T/C)[d]	p-value against N2[e]	p-value against specific control[f]
Fig. 4A	N2	20°C	20/29.5	20.5±0.5	64/2		
	gcn-2(ok871)	>>	21/31.4	20.67±0.33	116/4	0.7592	
	gcn-2(ok886)	>>	20/29.3	20.33±1.2	101/2	0.5729	
	atf-5(ok576)	>>	19/26.6	18.17±0.6	100/2	0.0117	
	N2	20°C	24/32.4	24.17±0.44	85/5		
	gcn-2(ok871)	>>	23/31.9	23±0.57	78/9	0.4991	
	gcn-2(ok886)	>>	23/29.8	23.17±0.61	77/2	0.0219	
	atf-5(ok576)	>>	22/28.4	22.33±0.33	109/5	0.0001	
	N2	20°C	21/28.6	21±0.67	82/12		
	gcn-2(ok871)	>>	22/27.9	22±1.00	83/13	0.9383	
Fig. 4C	*eat-2(ad465)*	20°C	24/33.3	23.5±0.5	71/3		
	gcn-2(ok871);eat-2(ad465)	>>	19/29.3	19.33±0.33	117/4	<0.0001	
Fig. 4B	N2/Control	20°C	24/32.2	24±1	80/4		
	N2/*gcn-2*(RNAi)	>>	25/32.8	25.33±0.33	92/2	0.1738	
	N2/*atf-5*(RNAi)	>>	23/30.7	23.25±0.75	84/1	0.0536	
Fig. S4 A	N2/Control	20°C/1mM	21/28.7	20.83±0.6	120/4		
	N2/*rrt-1*(RNAi)	>>	13/19.5	12.67±0.33	112/9	<0.0001	
	N2/*krs-1*(RNAi)	>>	12/14.5	11.67±0.33	114/2	<0.0001	
	gcn-2(ok871) /Control	>>	18/28.5	19±0.57	113/6	0.1614	
	gcn-2(ok871)/*rrt-1*(RNAi)	>>	12/19.3	12.67±0.33	122/12	<0.0001	0.1534
	gcn-2(ok871)/*krs-1*(RNAi)	>>	11/14.7	10.67±0.33	123/3	<0.0001	0.0009
	gcn-2(ok886)/Control	>>	17/29.4	17.67±0.88	114/7	0.2635	
	gcn-2(ok886)/*rrt-*	>>	13/20.1	12.5±0.28	120/6	<0.0001	0.6139

	1(RNAi)						
	gcn-2(ok880)/*krs-1*(RNAi)	>>	12/14.6	11.67±0.33	114/3	<0.0001	0.8192
Fig. S4 A	N2/Control	20°C/1mM	21/28.6	21.33±0.67	82/12		
	N2/*krs-1*(RNAi)	>>	12/14.4	12±0.0	96/9	<0.0001	
	gcn-2(ok871) /Control	>>	22/27.9	22±1	83/13	0.9383	
	gcn-2(ok871)/*krs-1*(RNAi)	>>	11/12.3	11±0.0	79/9	<0.0001	<0.0001
Fig. S4 B	N2/Control	20°C/0.25mM	19/26	19.83±0.44	61/4		
	N2/*rrt-1*(RNAi)	>>	16/25.3	15.67±0.33	131/4	<0.0001	
	gcn-2(ok871) /Control	>>	18/24.1	18.33±0.33	82/5	0.0510	
	gcn-2(ok871)/*rrt-1*(RNAi)	>>	14/23.2	13.83±0.16	135/3	<0.0001	0.0005
	gcn-2(ok886)/Control	>>	18/23.4	17.83±0.44	56/3	0.0673	
	gcn-2(ok886)/*rrt-1*(RNAi)	>>	14/22.8	14.33±0.33	135/3	<0.0001	0.0006
Fig. S4 B	N2/Control	20°C/0.25mM	20/32.2	20.33±0.67	89/4		
	N2/*rrt-1*(RNAi)	>>	16/25.3	16.33±0.33	117/7		<0.0001
	gcn-2(ok871) /Control	>>	21/30.8	20.75±0.43	129/1	0.6456	
	gcn-2(ok871)/*rrt-1*(RNAi)	>>	15/21.8	15±0.57	99/19		<0.0001
	atf-5(ok576)/Control	>>	21/26.2	21.5±0.29	142/2	0.3275	
	atf-5(ok576)/rrt-1(RNAi)	>>	17/26	17.67±0.33	132/12		<0.0001

a: Data sets within each panel were done in parallel and statistical analysis were performed within the data set.
b: Max lifespan is the mean of the last 10% surviving worms
c: Mean lifespan and standart error of the mean (s.e.m.) of 2-4 plates
d:Total number (T) of dead and censored (C) worms/censored (C)
e: *p*-value from log rank test comparing a strain to N2 or N2/Control(RNAi)(<0.05 is considered statistically significant)
f: *p*-value from log rank test comparing a strain to a specific control stain (e.g. *gcn-2;pek-1* vs *gcn-2* or *gcn-2/rrt-1(RNAi)* vs N2/*rrt-1(RNAi)*)
