# Supplementary material for: The general control nonderepressible-2 kinase mediates stress response and longevity induced by target of rapamycin inactivation in Caenorhabditis elegans
Source: Aging Cell. 2013 Jun 28;12(5):742–51. doi: 10.1111/acel.12101 (PMC4225475; doi:10.1111/acel.12101)
Supplement: Supplementary file 2 [file acel0012-0742-sd2.docx]

**Supporting Information**

**Fig. S1**

**

**

**Fig. S1**: Inactivation of leucyl-tRNA synthetase *lrs-1* induces phospho-eIF2α levels in a *gcn-2*-dependent manner. Western blot analysis showing the levels of phosphorylated eIF2α (P-eIF2α), normalized by the total amount of eIF2α, in whole extracts of 1-day N2 and *gcn-2(ok871)* worms, fed RNAi bacteria harboring the empty vector (Control) or expressing dsRNA for *lrs-1*.

**Fig. S2**


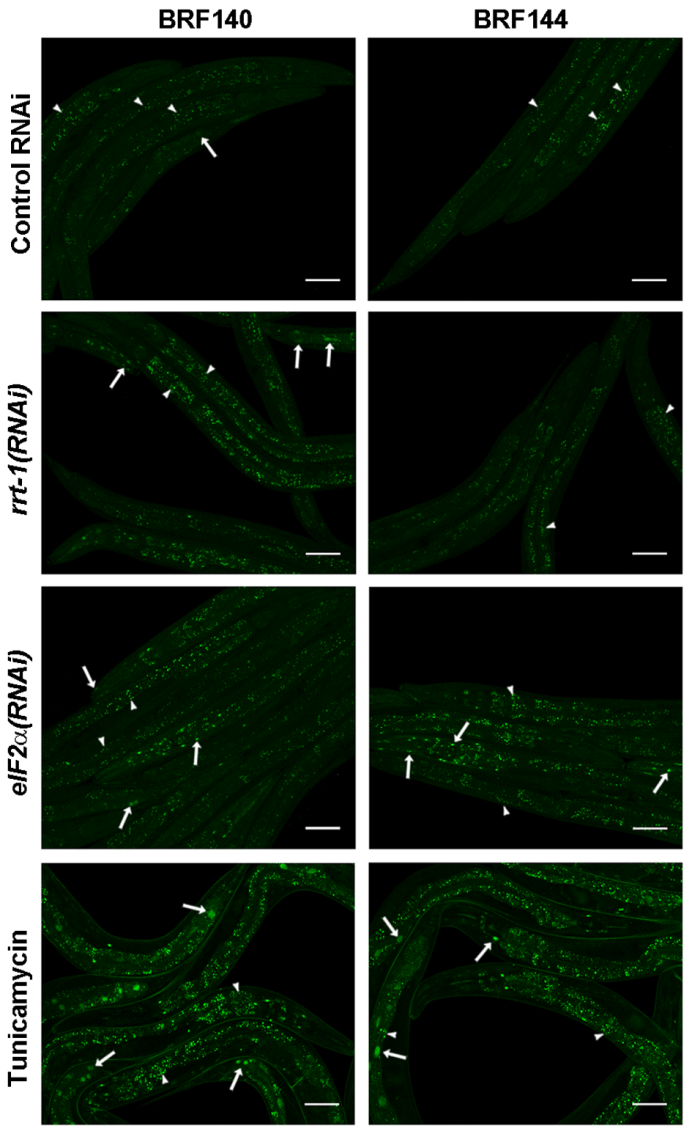


**Fig. S2**: Induction of *atf-5::gfp* transgene by *eIF2α(RNAi)* or tunicamycin does not require GCN-2 activity. Confocal images of adult transgenic worms BRF140 (N2Ex[ATF-5::GFP; pRF4]) and BRF144 (*gcn-2(ok871)*Ex[ATF-5::GFP; pRF4]) that were fed Control, *rrt-1(RNAi)* or *eIF2α(RNAi)* bacteria, or were treated with tunicamycin (5μg/ml for 24h). White arrows indicate fluorescent nuclei and white arrowheads show regions of autofluorescence. All images were taken at 20x magnification (scale bar: 50μm).

**Fig. S3**





**Fig. S3**: *gcn-2* deletion does not alter fertility in wild-type or *eat-2* mutants. Brood size (mean ± SD) of 5-10 individuals of the indicated strains at 20^o^C: **(A)** N2, *gcn-2(ok871)*, *gcn-2(ok886)* and *atf-5(ok567)*. **(B)** N2, *eat-2* and *eat-2;gcn-2* mutant animals.The asterisks represent statistical significant difference from N2 (***p<0.001 in unpaired t-test).

**Fig. S4**


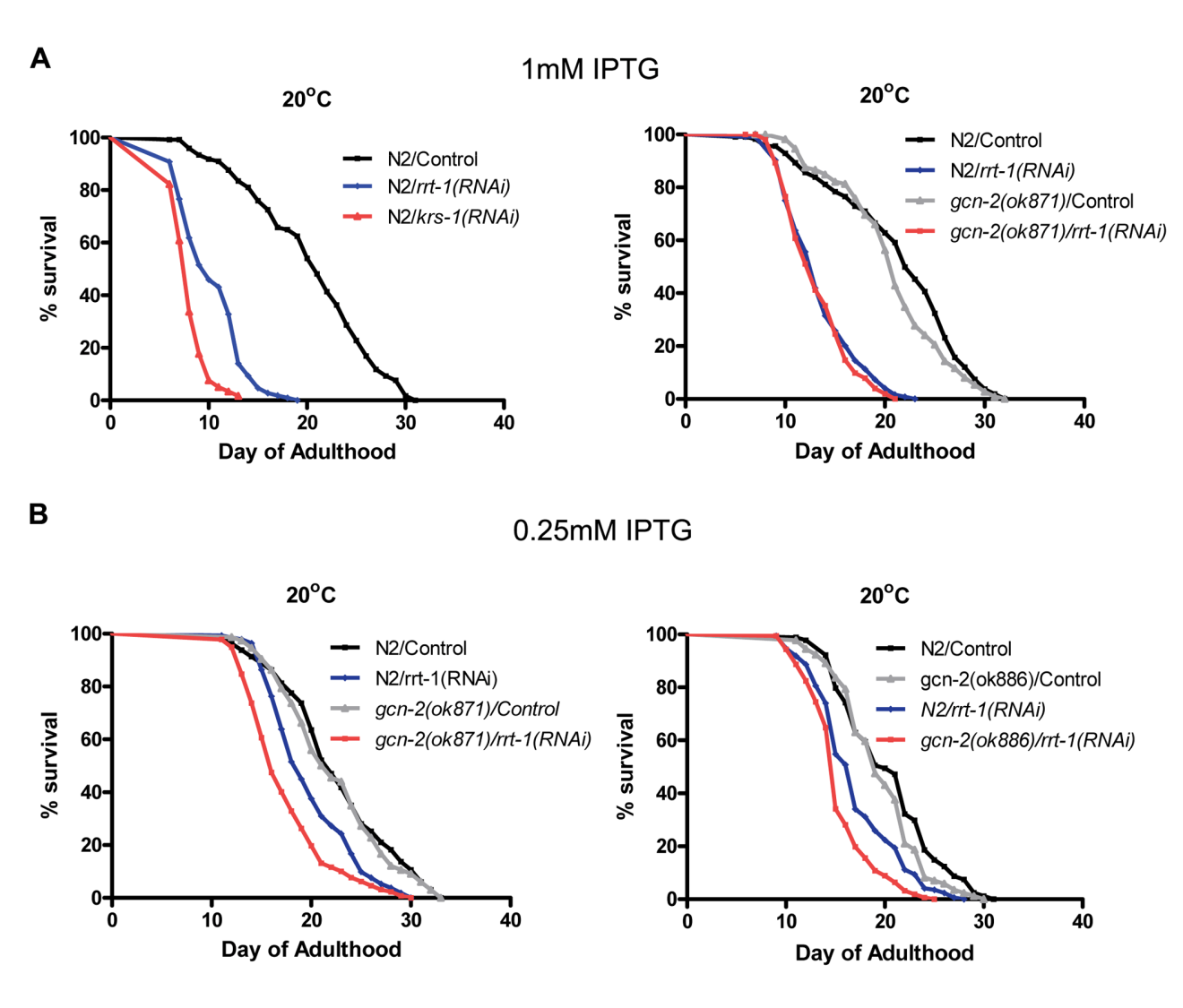


**Fig. S4**: Loss of GCN-2 sensitizes animals to amino acid limitation. **(A)** Survival curves of N2 and *gcn-2* mutant worms subjected to strong RNAi conditions (1mM IPTG) to inactivate *rrt-1* or *krs-1*, compared to empty vector (Control), at 20^o^C. **(B)** Survival curves of N2 and *gcn-2* mutant worms subjected to weak RNAi conditions (0.25mM IPTG) to inactivate *rrt-1* gene, compared to empty vector (Control), at 20^o^C. See Table 2 for additional data.

**Fig. S5**





**Fig. S5**: RNAi efficiency is not affected in *gcn-2* mutant worms. qRT-PCR of *let-363* transcript on N2 and *gcn-2* worms, subjected to Control RNAi or *let-363(RNAi)* expressing bacteria for 4 days. The asterisks represent statistical significant difference (*p<0.05, **p<0.01, ***p<0.001 in unpaired t-test).

**Fig. S6**





**Fig. S6**: GCN-2 regulates the induction of PHA-4 target genes in *eat-2* mutants. qRT-PCR of *lgg-1* and *mtl-1* transcript levels in 1-day adults of N2, *eat-2* and *eat-2;gcn-2* raised on OP-50 bacteria at 20^o^C. The asterisks represent statistical significant difference from N2 (*p<0.05, **p<0.01, ***p<0.001 in unpaired t-test)

**Fig. S7**


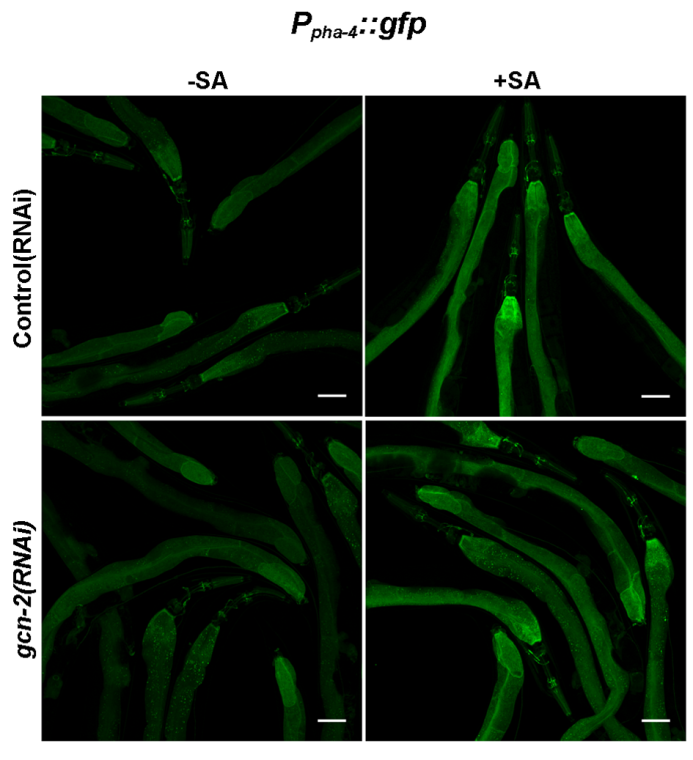


**Fig. S7**: Inactivation of *gcn-2* affects the induction of a P*_pha-4_::gfp* reporter under oxidative stress. Confocal images of 1-day adults expressing a membrane-bound GFP under the *pha-4* promoter (SM481strain), fed for two generations either Control RNAi or *gcn-2(RNAi)* expressing bacteria and treated (+SA) or not (-SA) with sodium arsenite (15mM for 3h before observation). All images were taken at 20x magnification under the same microscopy settings (scale bar: 50μm).

**Fig.S8**





**Fig. S8:** Loss of *gcn-2* but not *atf-5* increases the stress sensitivity of worms. **(A)** Survival of 1-day old N2, *gcn-2* and *atf-5* adults subjected to heat-shock (35^o^C for 6h). **(B)** Survival of 5-day old N2, *gcn-2* and *atf-5* adults after UV-irradiation (0.2J/cm^2^).

**Table S1: List of the strains used in this study**

| **Strain** | **Genotype** | **Information** | **Reference** |
| --- | --- | --- | --- |
| N2 |  | Wild-type |  |
| BRF162 | *gcn-2(ok871 II)* | Backcrossed to N2 4x | This study |
| BRF163 | *gcn-2(ok881 II)* | Backcrossed to N2 4x | This study |
| BRF172 | *atf-5(ok576 X)* | Backcrossed to N2 4x | This study |
| DA465 | *eat-2(ad465 II)* | Point mutation of Y48B6A.4. Backcrossed to N2 1x | CGC |
| BRF178 | *gcn-2(ok871I);eat-2(ad465 II)* | From crossing of BRF162 with DA465 | This study |
| BRF140 | N2 Ex[ATF-5::GFP;pRF4 *rol-6(su1006)*] | Translational fusion of the intact gene T04C10.4 under its promoter | This study |
| BRF144 | *gcn-2(ok871)*Ex[ATF-5::GFP;pRF4 *rol-6(su1006)*] | From crossing of BRF140 with BRF162 | This study |
| BRF152 | N2 Ex[_-uORFs_ATF-5::GFP;pRF4 *rol-6(su1006)*] | Translational fusion of the uORF-less gene T04C10.4 under its promoter | This study |
| SM481 | pxIs10[P*_pha-4_*::GFP::CAAX ; pRF4 *rol-6(su1006)*] | Membrane-bound GFP under the *pha-4* promoter | CGC |
| KX15 | *ife-2(ok306 X)* | Deletion of R04A9.4 (*ife-2*) | CGC |

**Table S2: List of primers used in this study**

| **Primer** | **Sequence (5' to 3')** | **Used for** |
| --- | --- | --- |
| GCN-2/1 (G1) | GCGATTGATGTTGTTCCAG | *gcn-2(ok871* and *ok886)* genotyping |
| GCN-2/2 (G2) | GAGACCACATCCATCGC | >> |
| GCN-2/3 (G3) | GTGAGTAGACTCGTCCG | >> |
| ATF-5/1 (A1) | ACATGGCATGCATGATTTATAACGGAAGTTCAG | *atf-5(ok576)* genotyping |
| ATF-5/2 (A2) | GGTCTAGAAATTTTCAGATGAGATGTTTCTGCG | >> |
| Eat-2/1 (E1) | GCTAGTCGATTTTCATCATCG | *eat-2(ad465)* genotyping |
| Eat-2/2 (E2) | GGCTAACCTTCAAATAGCAAAC | >> |
| GCN-2/4 (G4) | CTACCTACTCTCGAGTTCC | *gcn-2(RNAi)* construct |
| GCN-2/6 (G6) | AACTGCAGCTCATTGCTTCCAGCG | >> |
| ATF-5/3 (A3) | AACTGCAGAGTCGTCTCCCTTTCCTC | *atf-5(RNAi)* construct |
| ATF-5/4 (A4) | CGGGATCCGTCGGTGACAGTTTTCATTC | >> |
| RRT-1/1 (R1) | GGCAGATCTGGATACTCTGACTACTCAG | *rrt-1(RNAi)* construct |
| RRT-1/2 (R2) | GGCCTGCAGCAGCTTCACAACATACTCG | >> |
| KRS-1/1 (K1) | GCGCCATGGCAAGCCAAGAAGGAACAAG | *krs-1(RNAi)* construct |
| KRS-1/2 (K2) | GGGCTGCAGCGAGTGGTGACATGATTTG | >> |
| ATF-5/1 (A1) | ACATGGCATGCATGATTTATAACGGAAGTTCAG | *P_atf-5_::atf-5::gfp* construct |
| ATF-5/4 (A4) | CGGGATCCGTCGGTGACAGTTTTCATTC | >> |
| ATF-5/2( A2) | GGTCTAGAAATTTTCAGATGAGATGTTTCTGCG | *P_atf-5_::gfp* construct |
| ATF-5/5 (A5) | GGTCTAGAatggcttatgtaaatgaacaaaatcc | *_-uORF_::atf-5::gfp* construct |
| LRS-1/1 (L1) | GGCAAGCTTCAAGGATGATAAGGGAACTGG | *lrs-1(RNAi)* construct |
| LRS-1/2 (L2) | GGCCTCGAGCACGAAGCATCTGTCATCTG | >> |
| PHA-4/1 (P1) | CCCAAGCTTGCGGTCATCGGAAGAAGC | *pha-4(RNAi)* construct |
| PHA-4/2 (P2) | CCGCTCGAGCTGGTATACTCCGTTGGTG | >> |

**Table S3: Summary of Data from independent repeats of lifespan assays**

|  | Strain/RNAi | Treatment (Temp/IPTG) | Median/Max Lifespan (days)^b^ | Mean lifespan ± s.e.m (days)^c^ | Number (T/C)^d^ | p-value against N2^e^ | p-value against specific control^f^ |
| --- | --- | --- | --- | --- | --- | --- | --- |
| Fig. 4A | N2 | 20^o^C | 20/29.5 | 20.5±0.5 | 64/2 |  |  |
|  | *gcn-2(ok871)* | >> | 21/31.4 | 20.67±0.33 | 116/4 | 0.7592 |  |
|  | *gcn-2(ok886)* | >> | 20/29.3 | 20.33±1.2 | 101/2 | 0.5729 |  |
|  | *atf-5(ok576)* | >> | 19/26.6 | 18.17±0.6 | 100/2 | 0.0117 |  |
|  | N2 | 20^o^C | 24/32.4 | 24.17±0.44 | 85/5 |  |  |
|  | *gcn-2(ok871)* | >> | 23/31.9 | 23±0.57 | 78/9 | 0.4991 |  |
|  | *gcn-2(ok886)* | >> | 23/29.8 | 23.17±0.61 | 77/2 | 0.0219 |  |
|  | *atf-5(ok576)* | >> | 22/28.4 | 22.33±0.33 | 109/5 | 0.0001 |  |
|  | N2 | 20^o^C | 21/28.6 | 21±0.67 | 82/12 |  |  |
|  | *gcn-2(ok871)* | >> | 22/27.9 | 22±1.00 | 83/13 | 0.9383 |  |
| Fig. 4C | *eat-2(ad465)* | 20^o^C | 24/33.3 | 23.5±0.5 | 71/3 |  |  |
|  | *gcn-2(ok871);eat-2(ad465)* | >> | 19/29.3 | 19.33±0.33 | 117/4 | <0.0001 |  |
| Fig. 4B | N2/Control | 20^o^C | 24/32.2 | 24±1 | 80/4 |  |  |
|  | N2/*gcn-2*(RNAi) | >> | 25/32.8 | 25.33±0.33 | 92/2 | 0.1738 |  |
|  | N2/*atf-5*(RNAi) | >> | 23/30.7 | 23.25±0.75 | 84/1 | 0.0536 |  |
| Fig. S4 A | N2/Control | 20^o^C/1mM | 21/28.7 | 20.83±0.6 | 120/4 |  |  |
|  | N2/*rrt-1*(RNAi) | >> | 13/19.5 | 12.67±0.33 | 112/9 | <0.0001 |  |
|  | N2/*krs-1*(RNAi) | >> | 12/14.5 | 11.67±0.33 | 114/2 | <0.0001 |  |
|  | *gcn-2(ok871)* /Control | >> | 18/28.5 | 19±0.57 | 113/6 | 0.1614 |  |
|  | *gcn-2(ok871)*/*rrt-1*(RNAi) | >> | 12/19.3 | 12.67±0.33 | 122/12 | <0.0001 | 0.1534 |
|  | *gcn-2(ok871)*/*krs-1*(RNAi) | >> | 11/14.7 | 10.67±0.33 | 123/3 | <0.0001 | 0.0009 |
|  | *gcn-2(ok886)*/Control | >> | 17/29.4 | 17.67±0.88 | 114/7 | 0.2635 |  |
|  | *gcn-2(ok886)*/*rrt-1*(RNAi) | >> | 13/20.1 | 12.5±0.28 | 120/6 | <0.0001 | 0.6139 |
|  | *gcn-2(ok880)*/*krs-1*(RNAi) | >> | 12/14.6 | 11.67±0.33 | 114/3 | <0.0001 | 0.8192 |
| Fig. S4 A | N2/Control | 20^o^C/1mM | 21/28.6 | 21.33±0.67 | 82/12 |  |  |
|  | N2/*krs-1*(RNAi) | >> | 12/14.4 | 12±0.0 | 96/9 | <0.0001 |  |
|  | *gcn-2(ok871)* /Control | >> | 22/27.9 | 22±1 | 83/13 | 0.9383 |  |
|  | *gcn-2(ok871)*/*krs-1*(RNAi) | >> | 11/12.3 | 11±0.0 | 79/9 | <0.0001 | <0.0001 |
| Fig. S4 B | N2/Control | 20^o^C/0.25mM | 19/26 | 19.83±0.44 | 61/4 |  |  |
|  | N2/*rrt-1*(RNAi) | >> | 16/25.3 | 15.67±0.33 | 131/4 | <0.0001 |  |
|  | *gcn-2(ok871)* /Control | >> | 18/24.1 | 18.33±0.33 | 82/5 | 0.0510 |  |
|  | *gcn-2(ok871)*/*rrt-1*(RNAi) | >> | 14/23.2 | 13.83±0.16 | 135/3 | <0.0001 | 0.0005 |
|  | *gcn-2(ok886)*/Control | >> | 18/23.4 | 17.83±0.44 | 56/3 | 0.0673 |  |
|  | *gcn-2(ok886)*/*rrt-1*(RNAi) | >> | 14/22.8 | 14.33±0.33 | 135/3 | <0.0001 | 0.0006 |
| Fig. S4 B | N2/Control | 20^o^C/0.25mM | 20/32.2 | 20.33±0.67 | 89/4 |  |  |
|  | N2/*rrt-1*(RNAi) | >> | 16/25.3 | 16.33±0.33 | 117/7 |  | <0.0001 |
|  | *gcn-2(ok871)* /Control | >> | 21/30.8 | 20.75±0.43 | 129/1 | 0.6456 |  |
|  | *gcn-2(ok871)*/*rrt-1*(RNAi) | >> | 15/21.8 | 15±0.57 | 99/19 |  | <0.0001 |
|  | *atf-5(ok576)*/Control | >> | 21/26.2 | 21.5±0.29 | 142/2 | 0.3275 |  |
|  | *atf-5(ok576)/rrt-1(RNAi)* | >> | 17/26 | 17.67±0.33 | 132/12 |  | <0.0001 |

a: Data sets within each panel were done in parallel and statistical analysis were performed within the data set.

b: Max lifespan is the mean of the last 10% surviving worms

c: Mean lifespan and standart error of the mean (s.e.m.) of 2-4 plates

d:Total number (T) of dead and censored (C) worms/censored (C)

e: *p*-value from log rank test comparing a strain to N2 or N2/Control(RNAi)(<0.05 is considered statistically significant)

f: *p*-value from log rank test comparing a strain to a specific control stain (e.g. *gcn-2;pek-1* vs *gcn-2* or *gcn-2/rrt-1(RNAi)* vs N2/*rrt-1(RNAi*)
